# The complex scenario of obesity, diabetes and hypertension in the area of influence of primary healthcare facilities in Mexico

**DOI:** 10.1371/journal.pone.0187028

**Published:** 2018-01-25

**Authors:** J. E. Alcalde-Rabanal, E. Orozco-Núñez, O. E. Espinosa-Henao, A. Arredondo-López, L. Alcayde-Barranco

**Affiliations:** Center for Health System Research / National Institute of Public Health. Cuernavaca, México; Dasman Diabetes Institute, KUWAIT

## Abstract

**Introduction:**

Among non-communicable chronic diseases (NCCD), diabetes and hypertension are the main cause of adult mortality worldwide. Among the members of the Organization for Economic Cooperation and Development, Mexico is first in prevalence of diabetes and second in obesity. To face this problematic situation of NCCDs the Ministry of Health declared a national epidemiological alert against the overweight, obesity and diabetes. The target of this study is to characterize the status of obesity, diabetes and hypertension in the adult population in the area of influence of primary health facilities located in high social marginality areas.

**Methods:**

We conducted a cross-sectional observational study and used a convenience sample. A survey was conducted on a population of 18 years old and above in four primary health facilities in four Mexican States. The survey explored sociodemographic characteristics, the presence of chronic diseases, the access to healthcare services, risk factors and life styles. We also applied a complementary questionnaire to 20% of the participants, in order to explore food consumption during the last week and physical activity (International Physical Activity Questionnaire). We based our analysis on descriptive statistics and logistic multivariate regression to analyze factors associated with diabetes and hypertension.

**Results:**

73% (n = 7531, CI 0.72–0.74) percent of the population reported being diabetic, hypertensive and/or overweight. The majority of them receive healthcare in public health services. People over 40 years old, are 11 times more probable of living with diabetes and 8.7 times more probable of living with hypertension. Both conditions affect mostly women, whose main activity is to be a housewife. People who have lunch and dinner out of home are more likely to develop diabetes. People who perform intense physical activity are less likely to live with hypertension.

**Conclusion:**

According to the self-report, more than 70% of adult population living in areas with high social marginality suffer diabetes, hypertension and obesity. However, the percentage of people who live with these chronic conditions and are not aware of them, is unknown. The Mexican health system needs a primary healthcare that emphasizes on health promotion, timely detection of risk factors of Non Communicable Diseases and prevention of their complications.

## Introduction

Among non-communicable chronic diseases (NCCD), diabetes and hypertension are the main cause of adult mortality worldwide. In 2012, these diseases represented 82% of premature deaths, 68% of total deaths and three fourths of these deaths occurred in low and middle income countries. The obesity prevalence doubled in the last three decades. In 2014 the prevalence was 10% among men and 14% among women. At this time, the hypertension prevalence was 22% and 10% in the case of diabetes [1]. Alarmed by the increasing prevalence of these NCCDs and their impact on the health system and the socio-economic development of nations [2], The World Health Organization (WHO) and The Pan American Health Organization (PAHO) have considered obesity as one of the main risk factors for developing diabetes, hypertension, coronary heart diseases and cerebrovascular accidents, as well as certain types of cancer (endometrium, breast and colon).

Among the members of the Organization for Economic Cooperation and Development (OECD) [3], Mexico is first in prevalence of diabetes (15.9%) and second in obesity (32.4%), both in adult population. According to the National Health and Nutrition Survey (ENSANUT, for its Spanish acronym) 2012, the prevalence of overweight and obesity in adults was 71.3% (women 73%, men 69.4%) [4]. Diabetes reached 9.17% (women 9.67%, men 8.60%) [5], and hypertension 31.5% (women 30.7%, men 32.3%) [5]. The attributable proportion of overweight causing diabetes was estimated on 39.8% and 48.5% for obesity [6]. To face this problematic situation of NCCDs the Ministry of Health declared a national epidemiological alert against the overweight, obesity and diabetes.

The increase of these NCCD can be explained, in part, by the nutrition transition phenomenon experienced by the country. This transition has several characteristics: 1) increase in the availability of processed foods at low cost with high fat, salt and sugar contents; 2) increase in the consumption of fast food and food prepared out of home; 3) reduction of the available time for preparing food; 4) increase of the industrialized food publicity/marketing and offer to the general public and 5) reduction in the physical activity of the urban population [7]. This scenario of chronic diseases represent important challenges for the Mexican Health System [8].

The estimated direct cost of medical healthcare for overweight and obesity related diseases (cardiovascular and cerebrovascular diseases, hypertension, several cancers and type 2 diabetes mellitus) in 2000 was 2.033 billion dollars (26.3 billion pesos). The annual cost of health caring for obesity, diabetes and cardiovascular diseases in 2012 was around 6.033 and 7.812 billion dollars (around 78 and 101 billion pesos). The indirect costs associated with the loss of productivity was estimated from 5.646 to 7.8 billion dollars (around 73 and 101 billion pesos) [9]. The direct cost estimated for 2017 will be around 6.027 billion dollars (78 billion pesos) and the indirect cost affecting families will reach 5.973 billion dollars (73 billion pesos) [10]. These costs represent an economic burden for the health system and families (pocket expenses).

The suitable control of NCCDs is essential to avoid complications that hinder people’s quality of life, affecting socioeconomic development. While in developed countries half of the patients with common NCCDs [11] are under an adequate metabolic control, in Mexico only one out of five diabetics [12,13] and half of hypertensive patients are under control [5,14]. In Mexico the magnitude of NCCDs demands urgent intervention. Health authorities have proposed many strategies among which we highlight: the National Prevention and Promotion in Health Strategy [12], the National Overweight, Obesity and Diabetes Prevention and Control Strategy and the National Agreement for Healthy Nutrition [10].

As part of these strategies, an attempt was made of trying to engage the government, civil society and social sectors to influence their determinants. In relation to the healthcare determinant, Mexico has a network of hospitals and primary care facilities. They belong to the Mexican Institute of Social Security (IMSS, for its Spanish initials), the Ministry of Health (SSa, for its Spanish initials), the Institute of State’s Workers Security and Social Services (ISSSTE, for its Spanish initials) and Mexican Petroleum (PEMEX, for its Spanish initials), among others. In this network, the primary healthcare has an important role in the prevention and control of these chronic diseases, their risk factors, and their complications [15].

The most successful prevention of NCCDs has been linked to risk factors control, timely delivery of drugs, early detection of complications and coordination between primary health facilities and the hospitals [16] for treatment and control. Nevertheless, there are multiple explanations of why NCCDs control is limited in primary healthcare facilities. We highlight the following: undiagnosed people living with NCCDs [17] in the area of influence or primary health facilities, inappropriate clinical practices of personnel regarding quality of care, weak selfcare commitment of people living with NCCDs, lack of equipment, supplies and drugs [18]. International expectations by 2025 hope that at least 80% of basic technologies and essential drugs to treat chronic diseases will be available [1]. This goal will be impossible to meet if we do not have primary care strengthening oriented strategies. Considering this just mentioned goal, the target of this article is to characterize the status of obesity, diabetes and hypertension in the adult population within the area of influence of primary health facilities located in areas with high social marginality in Mexico as an essential input to manage the risk factors and control of NCCDs

## Methods

### Ethical approval

This article is part of the research project “Prevention of risks and health damages related to diabetes and hypertension in the context of effective universal coverage”. This project was financed by the National Council of Science and Technology (CONACYT, for its Spanish acronym) of Mexico in 2013. The Ethics Committee of the National Institute of Public Health issued approval N° 1487 for the project on February 19, 2014.

### Study design

We conducted a cross-sectional observational study and used a convenience sample considering the following criteria: a) previous experience of the state in promoting initiatives on chronic diseases healthcare and b) the interest of decision makers to participate in strengthening the healthcare model of NCCDs. Four states were included: Hidalgo, Jalisco, Morelos and Yucatan: In each state, we selected one primary health facility (PHF) with the largest number of diabetic or hypertensive patients, located in high social marginality areas (according to the marginality index of the National Population Council) and interest of the staff to participate in the project.

### Sample

For a single PHF we had a georeferenced map on which appeared all households under their responsibility. We draw an spiral on the map, starting on the place where PHF is located. All households in the spiral were included until we reach 1,000 households. The people who were 18 years old and over were included in the study. Additionally, we explored food consumption and physical activity in a subsample of households corresponding to 20% of the original 1000 households. In one of five households the complementary questionnaire was applied

### Instruments

#### Main survey

We applied a survey containing 76 questions (S4 File), which explored: sociodemographic characteristics, family antecedents, presence of chronic diseases, risk factors, life styles, health insurance and access to healthcare services. In this questionnaire, participants chose a physical silhouette that considered best represented their own [19,20] from **Stunkard, Sorensen, and Schulsinger Scale** (1983), which has been validated in Mexico. Also they were asked if they have been diagnosed as diabetic or hypertensive.

#### Complementary questionnaire

We applied a semi-quantitative questionnaire to explore daily food consumption during the last week. This questionnaire has been validated for Mexican population and explores type of food and quantity consumed per day (cup, piece, slice etc). It included 106 food items, classified into twelve groups: vegetables, fruits, cereals with and without fat, corn products, meats, low fat dairy products, snacks, fats, beverages and sweeteners.

In order to explore physical activity, we used the International Physical Activity Questionnaire (IPAQ) in its short version [21]. It explores physical activity’s intensity (vigorous, moderate, low), frequency (days per week) and duration (hours/minutes per day). The IPAQ 2002 considers as vigorous activities those that require strong physical effort and make breathing stronger (lifting heavy objects, aerobics, fast pedaling). Moderate activities are those that require intermediate physical effort (lifting light objects, regular pedaling, playing tennis).

People were asked to participate by a member of the INSP staff explaining the purpose, the ethical approval and implications of participating.

### Analysis

#### Body composition

**Fig 1:**
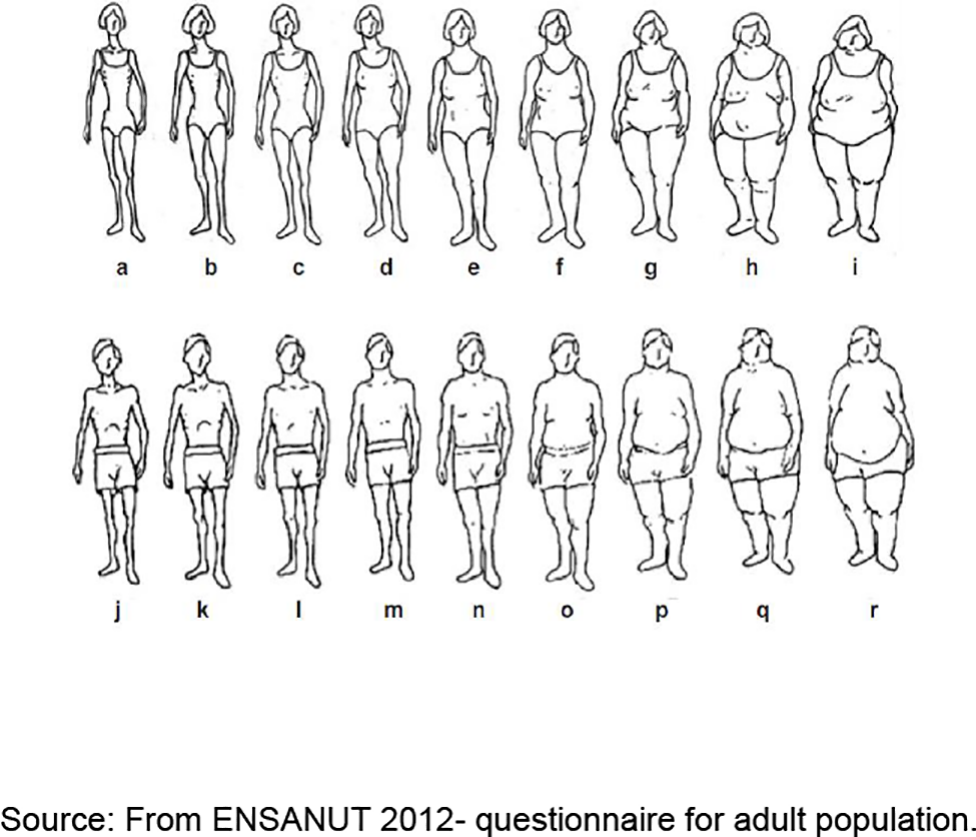
Stunkard, Sorensen, and Schulsinger Scale.

We estimated body composition using the Stunkard, Sorensen and Schulsinger Scale (1983) based on nine men and women figures (Fig 1). Women who chose figures a-c were classified as normal weight, those who chose figures d-f as overweight, and those who chose figures g-I were classified as obese. In the case of men, those who chose figures j-l were classified as normal weight, those who chose figures m-o as overweight, and those who chose figures p-r as obese.

#### Food consumption

According the type and quantity of food consumed per day, the nutrients (carbohydrates, protein, fiber and lipids) were estimated based on the Mexican Equivalences System (SMAE, for its Spanish initials) 2014 [22]. We excluded observations with daily consumption values below 500 kilocalories because OMS consider them as biologically not plausible. The same was done for observations over five standard deviations over the average consumption of total kilocalories consumed per day [23]. The cutoff points for calories, proteins, lipids, and fiber variables were established based on the Dietary Consumption References (DRI) and the adaptation of these references for the adult Mexican population [24].

#### Physical activity

We estimated the Metabolic Equivalent of Task (MET), as a physiological measure expressing the energy cost (or calories) of physical activities. One MET is the energy equivalent expended by an individual while seated at rest. While exercising, the MET equivalent is the energy expended compared to rest. So MET minutes are simply the time engaged in an activity: one minute of walking is equivalent to 3.3 METs, one minute of moderate physical activity is 4 METs and one minute of intense physical activity is 8 METs. The total of MET-min/week is the sum of hiking + moderate activity + vigorous activity. According IPAQ, the minimum acceptable activity per person is 10 minutes/day and its maximum is 180 minutes/day including walking, moderate and vigorous activity. To classify the activities we use the criteria shown in Table 1.

**Table 1:** Physical activity level–IPAQ 2002.

Intensity	Options	Description
High level	a)	Vigorous Activity at least 3 days (minimum total 1500 MET/week)
	b)	7 or more days of hiking, moderated activity and /or vigorous (minimum total of 3000 MET min/week)
Moderate level	a)	3 or more days of vigorous activity (minimum 20 min/day)
	b)	5 or more days of moderate activity and/or hiking 30 min/day
	c)	5 or more days hiking, moderate activity and/or vigorous minimum 600 METs-min/week
Low level	a)	Individuals who do not fulfill the high and moderate categories criteria

Adapted: Guidelness International Physical Activity Questionnaire (IPAQ)

We began by analyzing the data using descriptive statistics (frequency, means, etc.) to produce an overall picture. We estimate percentages as well as confidence intervals. To compare the characteristics of the sample with ones of the subsample, we analyzed some variables (state, gender, age, scholarship, health insurance, occupation, presence of chronic diseases and body composition) and chi^2^ test was performed. Finally, a logistic regression was developed to observe associated factors with diabetes and hypertension in marginalized areas. The Dependent variable were the presence of diabetes or hypertension and explanatory variables were gender, age, education, occupation, health insurance, body composition, place of food consumption, macronutrients consumption, physical activity and nutritional status. To test the regression model we estimated the goodness of fit.

## Results

We had information from 10,326 participants over 18 years old, 26% (n = 2 726) were from Hidalgo, 25% (n = 2 560) from Jalisco, 22% (n = 2 268) from Morelos and 27% (n = 2 762) from Yucatan. Male participants represented 47% (n = 4 830 = and 53% (n = 5 486) were females; 44% (n = 4 560) were over 40 years old. Concerning schooling, 9% (n = 958) had no education and over 60% (n = 6679) had basic education (elementary or secondary). The majority of the participants had a Public Health Insurance (SPS, for its Spanish initials) and IMSS. More than a third reported being employed at the moment of the survey and a bit more than a fourth of the participants were housewifes. In addition, 8.39% (n = 866) of the participants reported being diabetic, while 11.14% (n = 1149) reported being hypertensive and 64.65% (n = 6771) recognized having a wide abdominal circumference, which placed them in the overweight or obesity groups (Table 2).

**Table 2:** Comparative analysis of sample and subsample characteristics.

Variables	Main Sample (n = 10,316)		Sub-sample (n = 763)		P-value *
	n	Percentage (95% CI)	n	Percentage (95% CI)	
**State**					
Hidalgo	2726	26.42 (0.25–0.27)	193	25.29 (0.22–0.29)	>0.05
Jalisco	2560	24.82 (0.23–0.26)	172	22.54 (0.20–0.26)	>0.05
Morelos	2268	21.99(0.21–0.23)	193	25.29 (0.22–0.28)	>0.05
Yucatán	2762	26.77 (0.26–0.28)	205	26.87 (0.24–0.30)	>0.05
**Gender**					
Male	4830	46.82(0.46–0.48)	218	28.57 (0.25–0.32)	<0.05
Female	5486	53.18(0.52–0.54)	545	71.43 (0.68–0.75)	<0.05
**Age**					
Less than 40 years	5755	55.79 (0.55–0.56)	356	46.66 (0.43–0.50)	<0.05
More than 40 years	4560	44.21 (0.43–0.45)	407	53.34 (0.50–0.55)	<0.05
**Education**					
No education	958	9.29 (0.09–0.10)	65	8.52 (0.07–0.09)	>0.05
Basic education	6679	64.74(0.64–0.66)	536	70.25 (0.67–0.73)	<0.05
High School	1845	17.88 (0.17–0.19)	107	14.02 (0.12–0.17)	<0.05
High education	834	8.08 (0.08–0.09)	55	7.21 (0.05–0.09)	>0.05
**Health insurance**					
IMSS	3416	33.11 (0.32–0.34)	241	31.59 (0.28–0.35)	>0.05
ISSSTE	314	3.04 (0.03–0.40)	19	2.49 (0.02–0.04)	>0.05
SPS	4366	42.32 (0.41–0.43)	376	49.28 (0.46–0.53)	<0.05
Sedena	23	0.22 (0.01–0.02)	2	0.26 (0.01–0.03)	>0.05
Private	352	3.41 (0.03–0.04)	21	2.75 (0.02–0.04)	>0.05
None	1845	17.88 (0.17–0.19)	104	13.63 (0.11–0.16)	<0.05
**Occupation**					
Worker	702	6.80 (0.06–0.07)	32	4.19 (0.03–0.06)	>0.05
Employee	3829	37.12 (0.36–0.38)	167	21.89 (0.19–0.25)	<0.05
Own business	1208	11.71 (0.11–0.12)	114	14.94 (0.12–0.18)	>0.05
Housewife	2867	27.79 (0.27–0.29)	330	43.25 (0.40–0.47)	<0.05
Unemployed	439	4.26 (0.04–0.05)	37	4.85 (0.03–0.07)	>0.05
Other	1271	12.32 (0.12–0.13)	83	10.88 (0.08–0.13)	>0.05
Chronic diseases**					
Diabetes	866	8.39 (0.08–0.09)	99	12.97 (0.11–0.15)	<0.05
Hypertension	1149	11.14 (0.11–0.12)	119	15.58 (0.13–0.18)	<0.05
Overweight-obesity	6771	65.64 (0.65–0.07)	485	63.59 (0.60–0.67)	>0.05
Without NCCD	2797	27.11 (0.26–0.28)	217	28.44(0.25–0.31)	>0.05
**Body composition**					
Normal ^1^	3545	34.37 (0.33–0.35)	217	28.44 (0.25–0.32)	<0.05
Overweight	5942	57.61 (0.56–0.59)	479	62.78 (0.59–0.66)	>0.05
Obesity	829	8.03 (0.08–0.09)	67	8.78 (0.07–0.11)	>0.05

* P-values were calculated using chi-square test to compare the proportion of the main sample and sub-sample

**A person could suffer more than one NCCD

A total 763 informants participated in the subsample. When we compare the characteristics of the main sample with sub-sample (Table 2) the confidence intervals (CI) do not overlap in the case of gender, age, education (basic education), SPS, occupation (employed and housewife) and presence of diabetes and obesity.

**Fig 2:**
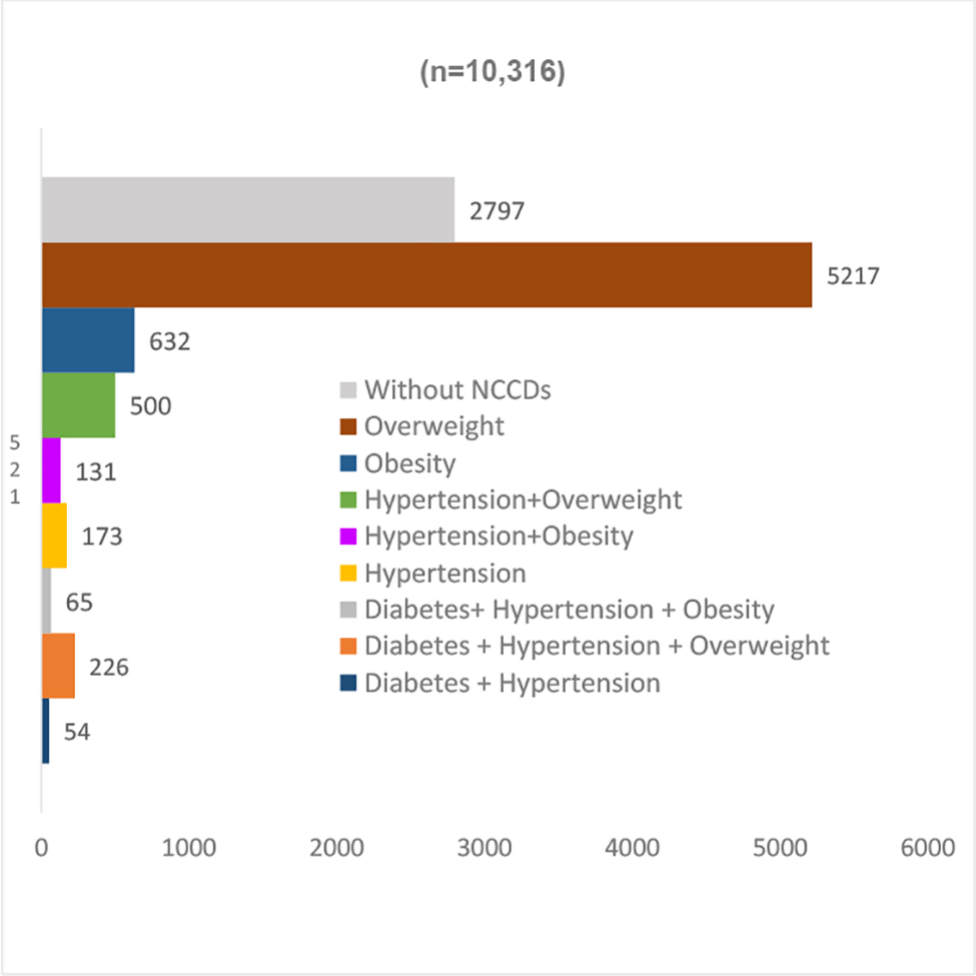
Population with chronic diseases in the area of influence of four primary health facilities. México 2014. N = 10,316.

**Fig 3:**
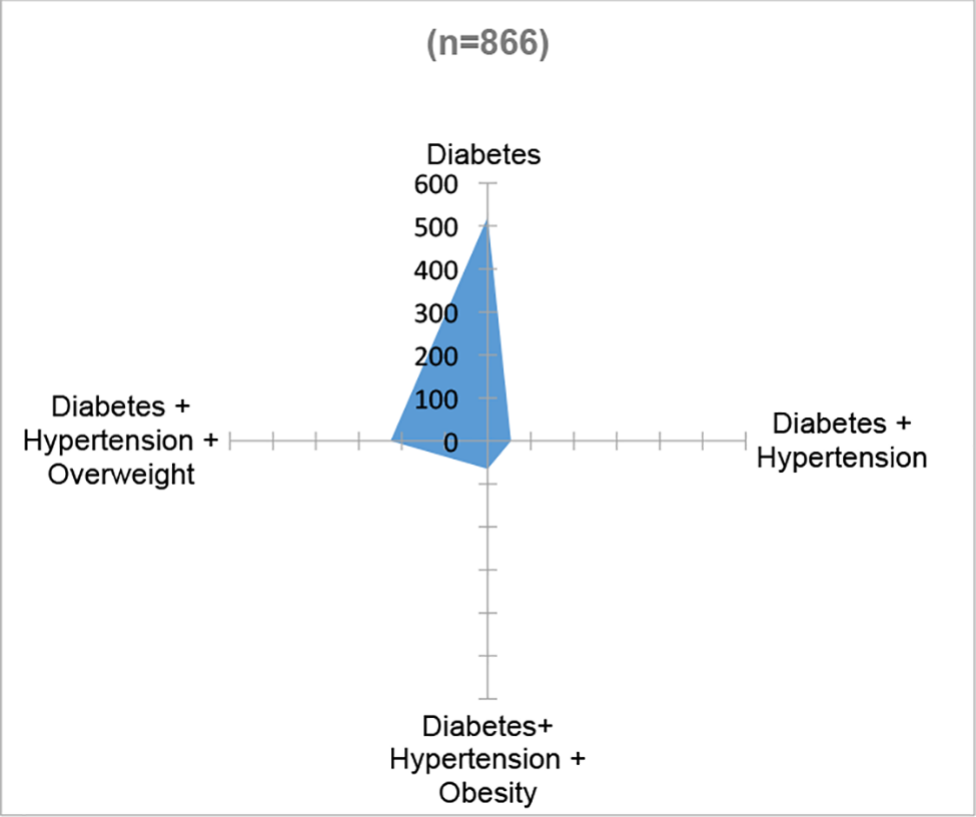
Comorbidity of diabetic population in the area of influence of four primary health facilities. México 2014.

**Fig 4:**
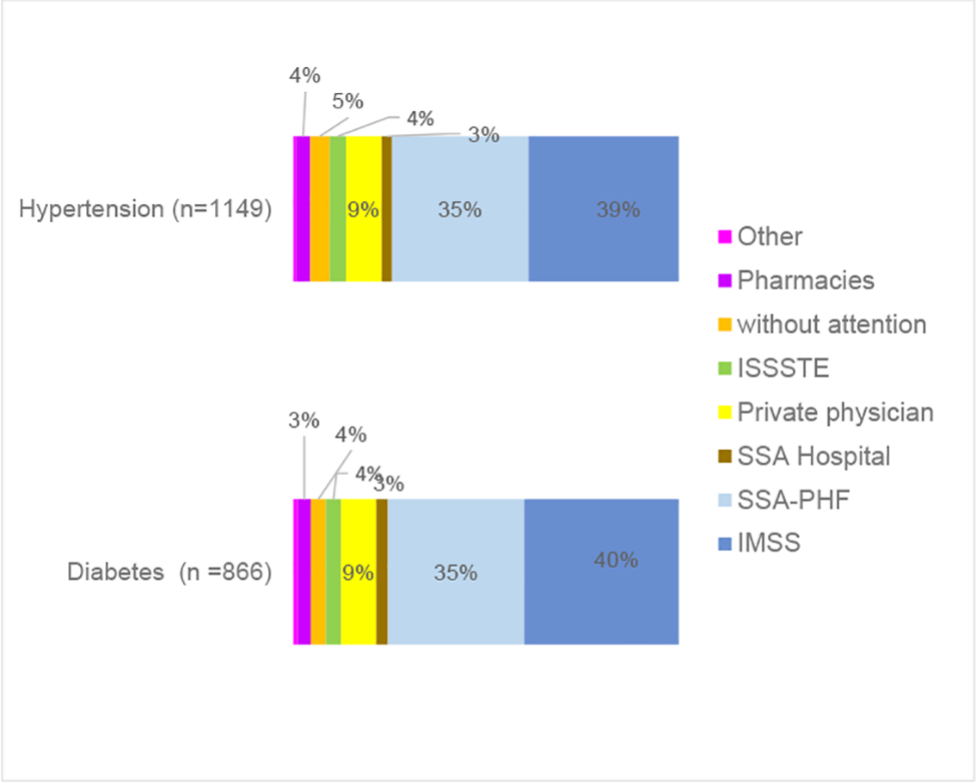
Providers of health care for diabetic and hypertensive population. México 2014.

The status of diabetes and hypertension in terms of incidence and risk factors is complex (Fig 2). Out of 10,326 participants, only 27% (n = 2,797; 95% CI 0.26 to 0.28) reported not having a chronic disease, 73% (n = 7,534; 95% CI 0.72 to 0.74) live with diabetes, hypertension and/or overweight or obesity. Among those who reported being diabetic (n = 866), 60% (n = 521; 95% CI 0.57 to 0.63) reported living only with this condition (Fig 3), while 6% (n = 54; 95% CI 0.5 to 0.08) reported also being hypertensive, 26% (n = 226; 95% CI 0.23 to 0.29) being diabetic, hypertensive and overweight, and 8% (n = 65; 95% CI 0.06 to 0.09) being diabetic, hypertensive and obese. In terms of the place where diabetics receive healthcare, 40% (n = 347; 95% CI 0.37 to 0.43) go to IMSS, 35% (n = 307; 95% CI 0.32 to 0.39) go to SSa clinics, 3% (n = 26; 95% CI 0.02 to 0.04) go to SSa hospitals, 9% (n = 79;; 95% CI 0.07 to 0.11) go to private physicians, 4% (n = 34; 95% CI 0.03 to 0.05) go to ISSSTE, 4% (n = 34; 95% CI 0.03 to 0.05) do not receive any healthcare, 3% (n = 29, 95% CI 0.02 to 0.04) go to pharmacies and 1% (n = 10) go to other healthcare provider (Fig 4).

**Fig 5:**
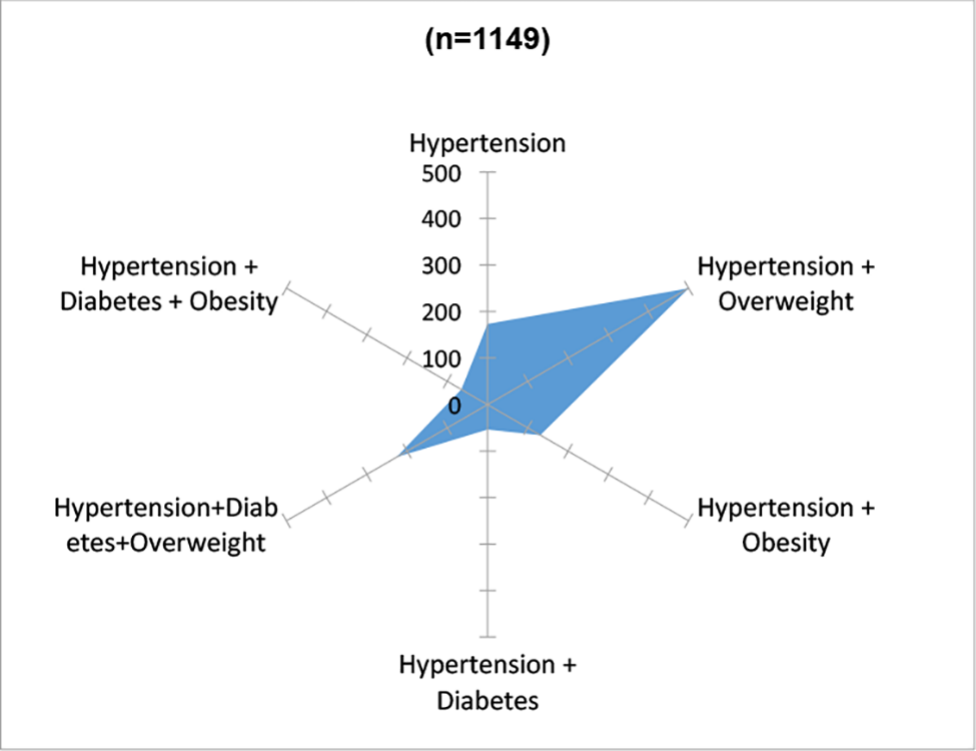
Comorbidity of hypertensive population in the area of influence of four primary health facilities. México 2014.

Among people who reported living with hypertension (n = 1,149), 15% (n = 173, 95% CI 0.13 to 0.17) only live with hypertension, 43.5% (n = 500, 95% CI 0.41 to 0.46) also is overweight, 11% (n = 131, 95% CI 0.09 to 0.13) live with hypertension and obesity, 5% (n = 54, 95% CI 0.03 to 0.06) with hypertension and diabetes and 25.5% (n = 291, 95% CI 0.23 to 0.28) live with hypertension, diabetes and overweight or obesity (Fig 5). The place where hypertensive patients receive healthcare, 39% (n = 448, 95% CI 0.36 to 0.42) go to IMSS, 35% (n = 407, 95% CI 0.33 to 0.38) go to SSa clinics, 3% (n = 31, 95% CI 0.02 to 0.04) go to SSa hospitals, 9% (n = 106, 95% CI 0.08 to 0.11) go to private physicians, 4% (n = 49, 95% CI 0.04 to 0.06) go to ISSSTE, 5% (n = 58, 95% CI 0.02 to 0.04) do not receive any healthcare, 4% (n = 42, 95% CI 0.03 to 0.05) go to pharmacies and 1% (n = 8) go to other provider (Fig 4).

**Fig 6:**
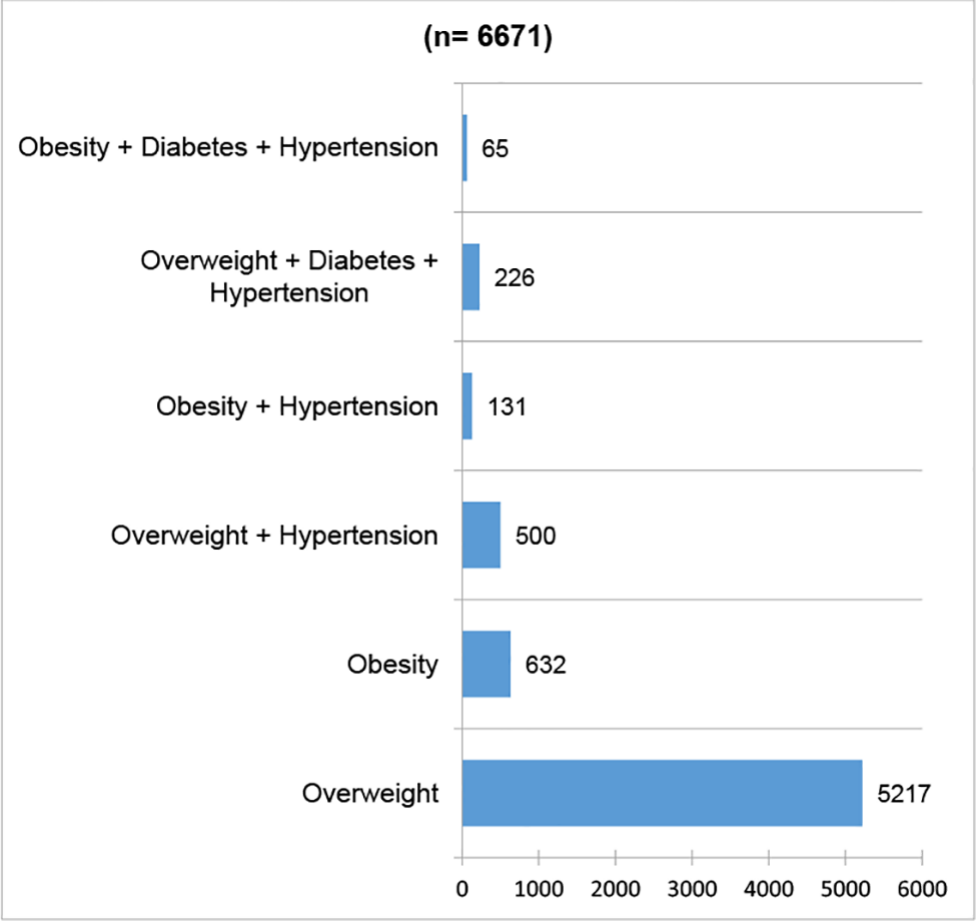
Comorbidity of overweight and obesity population in the area of influence of four primary health facilities. Mexico 2014.

Finally, of the people identified as overweight or obese (n = 6,671), 78% (n = 5,217, 95% CI 0.77 to 0.79) are overweight, 9% (n = 632, 95% CI 0.09 to 0.10) are obese, 7% (n = 500, 95% CI 0.07 to 0.08) are overweight and hypertensive, 3% (n = 131, 95% CI 0.01 to 0.02) are obese and hypertensive, 3% (n = 131, 95% CI 0.01 to 0.02) are overweight, diabetic and hypertensive and 1% is obese, diabetic and hypertensive (Fig 6).

### Associated factors of diabetes

The analysis of diabetes explanatory factors (R^2^ = 0.2597) did not show any differences between men and women (Table 3). Instead, we observed that people over 40 years old are 11 times more probable of living with diabetes than those under 40 are. We also observed that housewifes, unemployed, retirees and occasional workers, are between 2 to 5 times more probable of living with diabetes. People with higher education have 90% less probability of living with diabetes than those with no education (Table 3).

**Table 3:** Multivariate analysis of factor asociated to diabetes and hypertension. Mexico 2014.

Variables	Diabetes (n = 760)		Hypertension (n = 756)	
	Odds ratio (95% CI)*	P-value*	Odds ratio (95% CI)*	P-value*
**Gender**				
woman ^1^	1		1	
man	0.97 (0.47–1.99)	0.94	0.84 (0.45,1.56)	0.58
**Age**				
Less than 40^1^	1		1	
More than 40	11.2 (4.17–26.22)	<0.05	8.70 (4.24,17.83)	<0.05
**Education**				
No education	1		1	
Basic education	0.67 (0.34–1.30)	0.24	0.54 (0.28,1.03)	0.06
High School	0.41 (0.12–1.37)	0.15	0.25 (0.75,0.85)	0.03
High education	0.1 (0.01–1.04)	0.05	0.49 (0.13,1.83)	0.29
**Occupation**				
Employee^1^	1		1	
Own business	1.92 (0.70–5.27)	0.20	0.87 (0.36,2.11)	0.76
Homewife	2.20 (0.88–5.43)	0.09	1.49 (0.71,3.14)	0.07
Unemployed	5.30 (1.62–17.28)	0.01	1.42 (0.44,4.53)	0.56
Other	5.84 (2.18–15.57)	<0.05	2.89 (1.30,6.39)	0.01
**Health Insurance**				
SPS	1		1	
Uninsured	0.43 (0.16–1.17)	0.18	0.42 (0.16,1.017)	0.07
IMSS	1.46 (0.84–2.52)	0.71	1.38 (0.83,2.28)	0.21
Private	0.32 (0.04–2.71)	0.29	0.61 (0.12,3.03)	0.54
Other	1.35 (0.27–6.67)	0.10	1.42 (0.38,5.27)	0.60
**Body composition**				
Normal ^1^	1		1	
Overweight	1.31 (0.71–2.40)	0.37	1.61 (0.92, 2.83)	0.10
Obesity	1.66 (0.66–4.15)	0.28	1.69 (0.72,3.80)	0.20
**Place for food consumption**				
Home^1^	1		1	
Lunch out of home	2.56 (0.62–10.42)	0.19	4.23 (1.29,13.73)	0.02
No lunch	0.58 (0.26–1.29)	0.18	0.72 (0.35,1.49)	0.38
Meal out of home	1.16 (0.27–4.86)	0.84	4.02 (1.01,15.94)	0.05
Dinner out of home	0.16 (0.01–1.81)	0.14	1.1 (0.22,5.56)	0.90
No dinner	0.36 (0.12–1.06)	0.05	1.38 (0.61,3.09)	0.44
**Nutrients Consumption**				
Calories more than 2300 x day	1.27 (0.58–2.78)	0.55	0.58 (0.26,1.23)	0.16
Proteins more than 47 gr x day	2.21 (1.08–4.49)	0.03	0.76 (0.38,1.53)	0.45
Lipids more than 36 gr x day	1.54 (0.74–3.15)	0.24	1.65 (0.81,3.30)	0.17
Fiber more than 39.8 gr x day	0.70 (0.37–1.28)	0.25	1.09 (0.63,1.87)	0.76
**Physical activity**				
Low	1		1	
Moderate	0.7 (0.45–1.35)	0.24	0.79 (0.46,1.35)	0.40
Intense	0.61 (0.27–1.08)	0.20	0.49 (0.24,0.97)	0.04
**State**				
Hidalgo	1		1	
Jalisco	0.51 (0.23–1.10)	0.09	1.25 (0.63,2.46)	0.52
Morelos	0.65 (0.30–1.38)	0.26	0.69 (0.34,1.42)	0.32
Yucatan	0.84 (0.36–1.98)	0.69	0.94 (0.41,2.11)	0.87

1: reference category

* Odds rario and P-values were calculated using Multivariate logistic regresión model

We did not find any statistically significant coefficients related to health insurance, body composition. Nevertheless, overweight (OR = 1.31) and obese (OR = 1.66) people are more likely to live with diabetes than normal weight people are.

Interestingly, people who have lunch (OR = 2.56) or dinner (OR = 1.15) outside of home are more likely of being diabetic than those who eat these meals at home. Not having lunch (OR = 0.57) or supper at home (OR = 0.15) or not having supper at all (OR = 0.36) reduces the probability of being diabetic, even though these values are not statistically significant. Besides, people consuming more than 2,300 calories per day (OR = 1.27), more than 47 grams of proteins (OR = 2.20), more than 36 grams of lipids (OR = 1.53) are more likely to live with diabetes. Instead, those who consume more than 38.9 grams of fiber (OR = 0.69) are less likely to live with diabetes. We also did not observe differences in relation to physical activity, but coefficients show that increased physical activity is related to a lower probability of being diabetic.

### Associated factors of hypertension

The analysis of the hypertension explanatory factors of (R^2^ = 0.2285) showed no differences between men and women. Instead, we observed that people over 40 years old are 8.7 times more likely to live with hypertension than people under 40. In relation to occupation, those working at home (OR = 1.49) and those with another employment (retirees and part-time workers) (OR = 2.88) are more likely to live with hypertension. Overweight people are 61% more probable of being hypertensive than people with normal weight. People who have lunch (OR = 4.2) and dinner (OR = 4) out of home are more likely to live with hypertension than those eating at home (Table 3).

People with basic education (OR = 0.54) and with high school (OR = 0.25) are less likely to be hypertensive than people with no education. People with no health insurance (= R = 0.42) are more likely to be hypertensive than those with SPS. People who perform intense physical activity (OR = 0.48) are less likely to be hypertensive than those who perform low physical activity.

We did not find statistically significant coefficients in health insurance status, but coefficients show that a person with no health insurance (OR = 0.42) is less likely to be hypertensive than a person with SPS. Neither have we found any significant differences in body composition. Nevertheless, overweight (OR = 1.61) and obese people (OR = 1.69) are more likely be hypertensive than people with normal weight.

## Discussion

Results show a challenging NCCDs scenario in terms of opportune diagnosis and treatment. ENSANUT 2012 estimated that 9.1% of the population lived with diabetes, in our study found that 8.39% of the population living with this condition. Therefore, we can infer that there is a very small proportion of the people living with diabetes that did not report it. On the other hand, in 2016 OECD reported 15.9% prevalence of diabetes in Mexico, if we consider this percentage almost 50% of participants in our study living with this condition are unaware of it [18]. Since our estimation was based on self-report, we must stress out that we cannot know the exact percentage of people living with diabetes who are not aware of it.

In the case of hypertension, 11.64% of the study’s population, reported living with this condition; ENSANUT 2012 estimated that 31.5% of the population was hypertensive. This means that 20% of the population lives with hypertension without knowing about it (in 2012, 47.3% did not know themselves as being hypertensive) [25]. The unawareness of a subject living with NCCD is part of the explanation of late detection and treatment. Other studies have calculated that almost a third of the diabetics do not know it, so when the diagnostic arrives, they already present complications [26].

Out of the all participants in our study, 64.5% was classified as overweight or obese, while the prevalence of this conditions reported by ENSANUT 2012 was 71.28%. We may explain this difference because the self-recognize of silhouette and higher prevalence is observed in the North region (76.8, CI: 75.5–78.1) than the South region (71.3, CI:71.3–72.4;which includes Morelos and Yucatan) and the Center region (73.9 CI: 72.6–75.1; which includes Hidalgo and Jalisco) [27].

It is worrying to find out that 73% of the population above 18-year-old, lives with at least one chronic condition such as diabetes, hypertension, overweight or obesity. Among this percentage, 9% lives at least with 2 chronic conditions and 4% with 3 of them. According to WHO, an optimal health status for the population considers BMI values between 21–23 kg/m^2^. In the studied group, the average BMI was 27.16 kg/m^2^. If we consider overweight and obesity among the main risk factors, this population is in high risk of chronic diseases. The fact is that only one fourth of the studied population does not have any chronic disease or risk factor.

As far as the place where people with NCCDs received treatment, 75% reported being treated in public services (IMSS or SSa). This result was expected because health facilities were located in marginal areas where the population is usually registered in SPS or IMSS. This information is coherent with the one reported by ENSANUT 2012, which states that around 70% of diabetics and 90% of hypertensive patients report being insured by SPS or IMSS [5]. Another result deserving attention is that around 4% of the population living with NCCDs has no access to healthcare at all. This evidences the barriers to universal healthcare that the Mexican health system is still facing [5, 28].

Besides, 9% of the people who know they are diabetic and hypertensive receive care from private providers. This means that people with scarce resources incur in health pocket spending increasing their risk of catastrophic expenses and impoverishment conditions. In 2013, a systematic review reported that the proportion of households with chronically ill members in poverty conditions increased from 45% to 53% [29]. In Mexico, private out of pocket health expenditures is still high, reaching 43.98% in 2014 [30].

In relation to NCCDs associated factors, people over 40 years old are more likely of being diabetic or hypertensive because of the long term evolution of these conditions. The prevalence of this diseases is generally associated with adulthood [31,32,33]. Our study shows a bigger risk of living with diabetes among unemployed and other occupations (including those who work occasionally) high levels of stress have documented [34,35]. High levels of education are associated to a less probability of being diabetic. This study found a greater risk of being diabetic among people with low education levels; which is also associated with a less probability of being diagnosed and adhering to treatment [36].

One of the findings that attracted our attention is the greater probability of being diabetic and hypertensive among those people who have public health insurance. One possible explanation is that people with less resources who suffer from a chronic disease, deliberately search to enroll in public insurance schemes in order to have access to medical examination, laboratory tests, medicines and other things that their families would otherwise have to pay for. Instead, we found a less probability of being diabetic and hypertensive among people with no insurance at all. This may be associated to their healthy condition making them procrastinate their enrollment because they do not feel the need to look for it.

Among the studied population, we found a protector factor against diabetes to be a fiber rich diet. This is similar to what another study on diet patterns with high levels of fiber consumption, together with low levels of fat consumption, found as a factor notably reducing the incidence of diabetes. This means that changes in diet patterns have an important role in the etiology of diabetes with similar results in men and in women [37,38].

Considering life styles, people who have lunch or dinner out of home are more likely to be diabetic, which might be related to the fact that these diets are hyper-caloric and hyper fat containing, while their consumption is uncontrolled [39]. In the case of people who do not have supper, this means that there is a reduced consumption of hyper-caloric food. In this sense, there are recommendations on how to plan meals out of home and the consumption of a light supper can help to as prevent and control obesity as a diabetes risk factor [40]. In the case of hypertension, we observed that people who perform intense physical activity are less prone to suffer from this condition. While studies have documented that regular practice or physical exercises can reduce the incidence of hypertension [41,42].

The scenario of NCCDs becomes complicated, only 2 out of 10 people with diabetes mellitus and 5 out of 10 with hypertension have an adequate control [5]. In the meantime, it is necessary to improve the therapeutic adherence [43,44] of population’s life styles, quality and continuity of healthcare [12] self-care and co-responsibility of the people with chronic diseases. Interventions in promotion of healthy life styles and group work show a greater influence in reducing the progression of these diseases than pharmacotherapy [45,46]. Also, the proper performance of health facilities assures a higher number of consultations per chronic patient and a better metabolic control [46,47]. These figures show that the Mexican health system is facing a very big challenge concerning NCCDs that demands the strengthening of primary healthcare [48,49].

The main limitation of our study is the fact that all estimations are based on self-report of diabetes and hypertension; obviously, the prevalence of these diseases is underestimated. Another limitation is that we had to estimate the body composition from the self-perceived silhouette the participants better thought represented them, which might have led to misclassification. Additionally, we had a small sample size therefore the findings are not generalizable.

## Conclusion

This study found out that almost three fourths of the population over 18 years old living in the area of influence of the primary health facilities located in high social marginality areas, have at least one chronic disease like diabetes, hypertension, overweight or obesity. This is a worrying fact. If we consider that overweight and obesity are among the main risk factors for NCCDs, this population is in high risk of developing NCCDs. As this estimation was based on self-report of the participants, the real percentage of the population living with these NCCDs is unknown. These people have also not been identified by PHF in order to initiate timely treatment and prevent its complications.

The approach to these diseases requires strengthening of primary care in order to act upon the main risk factors such as overweight and obesity. Doing so will help avoiding early development of diabetes and hypertension cases. It is also essential to have efficient health intervention based on strategic programs to assure early detection of risk factors and prevention of these diseases and their complications. This is definitely one of the biggest challenges for the Mexican health system.

## Supporting information

S1 File Data for Fig 1. (PDF)

S2 File Data for Fig 1, Fig 2, Fig 3, Fig 4, Fig 5 and Table 2. (DTA)

S3 File Data for Table 2 and Table 3. (DTA)

S4 File English translated questionnaire. (DOCX)
