# Supplementary material for: The complex scenario of obesity, diabetes and hypertension in the area of influence of primary healthcare facilities in Mexico
Source: PLoS One. 2018 Jan 25;13(1):e0187028. doi: 10.1371/journal.pone.0187028 (PMC5784882; doi:10.1371/journal.pone.0187028)
Supplement: S1 File — (PDF) [file pone.0187028.s001.pdf]

Instituto Nacional de Salud Pública

SECRETARÍA DE SALUD

ENCUESTA NACIONAL DE SALUD Y NUTRICIÓN 2012

GOBIERNO FEDERAL SALUD

CUESTIONARIO DE ADULTOS DE 20 AÑOS O MÁS

1. IDENTIFICACIÓN GEOGRÁFICA

Resultados_Entrevistas_Adul

ENTIDAD FEDERATIVA desc_ent entidad

MUNICIPIO O DELEGACIÓN desc_mun munici

LOCALIDAD desc_loc locali

CLAVE DE AGEB.. ageb

MANZANA .... manzana

2. CONTROL DE CUESTIONARIO

HOGAR............... DE DE LA VIVIENDA

CUESTIONARIO DE DEL HOGAR

NÚMERO DE VIVIENDA vivcroq

FOLIO: folio

3 DIRECCIÓN DE LA PERSONA SELECCIONADA

Resultados_Entrevistas_Adul

calle

(CALLE, AVENIDA, CALLEJÓN, CARRETERA, CAMINO, BOULEVARD, KM.)

NOMBRE DEL JEFE DE FAMILIA

no_ext — NÚMERO EXTERIOR

no_int — NÚMERO INTERIOR

colonia — (COLONIA, FRACCIONAMIENTO, BARRIO, UNIDAD HABITACIONAL)

cp — CÓDIGO POSTAL

4. DATOS DEL ADULTO SELECCIONADO

Individual_Adultos

NÚMERO DE RESIDENTE DEL ADULTO SELECCIONADO intp

SEXO: asexo HOMBRE.......................1 MUJER.......................2 EDAD: AÑOS aedad MESES

5. RESULTADO DE LA VISITA

Resultados_Entrevistas_Adul

NÚMERO DE VISITA	1a.	2a.	3a.	4a.
NOMBRE Y CLAVE DEL ENTREVISTADOR	nom_e1	nom_e1	nom_e1	nom_e1
FECHA (dd mm aa)	vis1f	vis2f	vis3f	vis4f
RESULTADO (*)	vis1r	vis2r	vis3r	vis4r
HORA DE INICIO	vis1h	vis2h	vis3h	vis4h
HORA DE TÉRMINO	vis1ft	vis2ft	vis3ft	vis4ft

(*) CÓDIGO PARA EL RESULTADO DE LA VISITA

01 ENTREVISTA COMPLETA
02 ENTREVISTA INCOMPLETA
03 INFORMANTE INADECUADO
04 ENTREVISTA APLAZADA (HACER CITA)
05 AUSENCIA DE PERSONA SELECCIONADA EN EL MOMENTO DE LA VISITA
06 SE NEGÓ A DAR INFORMACIÓN
07 VIVIENDA DESHABITADA
08 VIVIENDA DE USO TEMPORAL
09 HABLA ALGUNA LENGUA EXTRANJERA
10 NO ES VIVIENDA
11 OTRO (ESPECIFICAR EN OBSERVACIONES)
99 ADULTO QUE SE CONVIERTE EN ADOLESCENTE

A. ¿Cuál es el sexo del(de la) entrevistado(a)? asexo

ENTREVISTADOR: ESTA PREGUNTA DEBE SER CONTESTADA POR USTED

Hombre...1

Mujer...2

B. ¿Cuántos años cumplidos tiene actualmente? aedad

Edad [___|___|___|

NS/NR..999

ENTREVISTADOR: ANTES DE INICIAR DÍGALE AL ADULTO QUE DURANTE LA ENTREVISTA PUEDE UTILIZAR LENTES Y/O APARATO(S) AUDITIVO(S) EN CASO DE QUE LOS USE HABITUALMENTE

ENTREVISTADOR:

14.0 El adulto presenta alguno de los siguientes problemas: a1400

Tiene problemas auditivos graves (sordo) 1

Tiene problemas para hablar o comunicarse 2

No presenta los problemas 1 y 2 3

FILTRO:

1.- PERSONAS DE 20 A 59 AÑOS CON CÓDIGO 1 y/o 2 EN 14.0 PREGUNTE POR EL INFORMANTE SUSTITUTO/PROXY Y PASE A 14a.11, CONTINÚE LA ENTREVISTA HACIENDO LAS PREGUNTAS SOBRE EL ADULTO AL INFORMANTE SUSTITUTO/PROXY

2.- PERSONAS DE 20 A 59 AÑOS CON CÓDIGO 3 EN 14.0 PASE A 0.1

3.- PERSONAS DE 60 AÑOS Y MÁS CON CÓDIGO 1 y/o 2 EN PREGUNTA 14.0, PREGUNTE POR EL INFORMANTE SUSTITUTO/PROXY Y PASE A 14a.11, CONTINÚE LA ENTREVISTA HACIENDO LAS PREGUNTAS SOBRE EL ADULTO MAYOR AL INFORMANTE SUSTITUTO/PROXY

4.- PERSONAS DE 60 AÑOS Y MÁS CON CÓDIGO 3 EN PREGUNTA 14.0 CONTINÚE

XIV A. ESTADO COGNOSCITIVO

A continuación le voy a presentar una serie de ejercicios para ver cómo funciona su memoria. Le voy a hacer algunas preguntas y le voy a pedir que realice algunas tareas. Puede sentir que algunas son más difíciles que otras. Si no puede contestar alguna pregunta o realizar una tarea, no se preocupe; lo importante es que realice su mejor esfuerzo y que me dé la mejor respuesta que pueda.

Queja subjetiva de memoria

14a.01 ¿Ha tenido dificultades con su memoria que sean un problema para usted? a14a01

No..1

Sí, de intensidad ligera/moderada o infrecuente...............2

Sí, de intensidad severa, frecuente o persistente.............3

La pregunta no se entendió, no se obtiene respuesta o es inaudible, inapropiada, incoherente, el valor es incierto..4

Dato no obtenido, pregunta no realizable o no aplicable..9

Mini-COG

14a.03 Ahora le voy a pedir que repita tres palabras y trate de memorizarlas porque se las voy a preguntar más adelante. Las palabras son: árbol, casa, perro. Repítalas

ENTREVISTADOR: COLOQUE "1" POR CADA PALABRA REPETIDA Y "0" SI NO REPITIÓ LA PALABRA

**Palabra**	**Correcto**	
Árbol	[____]	a14a03a
Casa	[____]	a14a03b
Perro	[____]	a14a03c
**Total**	**[____]**	

Fluencia verbal (número de animales en 1 minuto)

Instrucciones:

"Quiero que piense en todos los animales que conoce que vivan en el aire, en el agua, en la tierra, en el bosque, todas las clases de animales. Y que me diga todos los nombres de los animales que recuerde, EN UN MINUTO. ¿Está listo? Vamos a comenzar."

ENTREVISTADOR: CUENTE LOS ANIMALES MENCIONADOS, EN UN MINUTO, POR EL ADULTO MAYOR SELECCIONADO. NO AYUDE NI DÉ PISTAS.

14a.03a Número de animales evocados: |____|____| a14a03_a

Individual_Adultos

XIV A. ESTADO COGNOSCITIVO

Reloj

14a.04 Ahora le voy a mostrar UN CÍRCULO EN EL CUAL VA A DIBUJAR la figura de un reloj. Le voy a pedir por favor que dibuje la carátula con todos sus números y las manecillas marcando las 11:10 (Once y diez).

ENTREVISTADOR: LA PRUEBA DEBE REALIZARSE EN UN TIEMPO MÁXIMO DE 3 MINUTOS. NO AYUDE NI DÉ PISTAS.

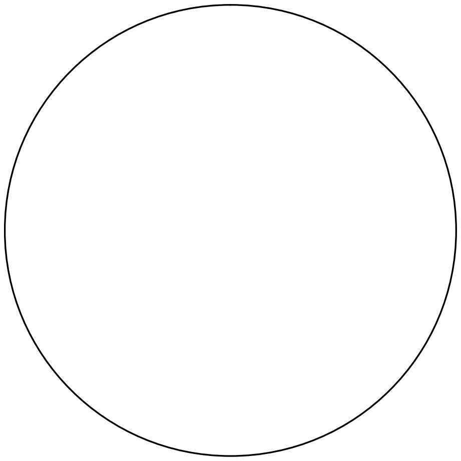

Mini-COG

14a.05 La prueba no se realizó porque el adulto mayor tiene dificultad para escribir debido a que: a14a05

Presenta problemas de visión..1
Presenta problemas motores y/o articulares....................2
No presenta alguno de los problemas anteriores y se negó a hacer la prueba...3
→ PASE A 14a.09
Realizó la prueba...4

Permítame un momento, anotaré algunos datos sobre el ejercicio realizado.

ENTREVISTADOR: CALIFIQUE DE ACUERDO A LOS SIGUIENTES CRITERIOS, GUIÁNDOSE POR EL MODELO

UTILICE ESTA FIGURA COMO GUÍA PARA CALIFICAR LAS PREGUNTAS 14a.06, 14a.07 y 14a.08

1 **Manecilla corta (horas)**

2 **Manecilla larga (minutos)**

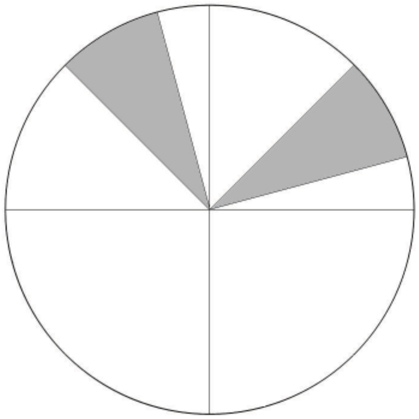

14a.06 ¿Se encuentran ubicados los números correspondientes a un reloj analógico (del 1 al 12) dentro de la carátula del reloj en la secuencia correcta? a14a06

Sí...1
No..2

14a.07 Imaginando cuatro partes iguales (cuadrantes) dentro de la carátula del reloj, ¿alguno de los cuadrantes contiene más de tres números? a14a07

Sí...1
No..2

14a.08 ¿Las manecillas se encuentran ubicadas, de manera aproximada, dentro del área sombreada correspondiente? a14a08

Sí...1
No..2

14a.09 ¿Recuerda que le leí en voz alta una lista de palabras? Por favor diga nuevamente las 3 palabras que le mencioné.

ENTREVISTADOR: PERMITA EL RECORDATORIO ESPONTÁNEO, NO AYUDE A LA PERSONA MAYOR.

COLOQUE "1" POR CADA PALABRA REPETIDA Y "0" SI NO REPITIÓ LA PALABRA.

EL TIEMPO MÁXIMO PERMITIDO ES DE UN MINUTO

**Palabra**	**Correcto**	
Árbol	[____]	a14a09a
Casa	[____]	a14a09b
Perro	[____]	a14a09c
**Total**	**[____]**	

ENTREVISTADOR:

14a.10 Si el adulto mayor no recuerda ninguna de las palabras de la pregunta 14a.09, es decir, el total fue "0 puntos", se solicitará un informate sustituto/proxy. a14a10

Si la suma es 1 o más................................1 → PASE A 14b.01

Si la suma es 0................................2 → PREGUNTE POR EL INFORMANTE SUSTITUTO Y CONTINÚE LA ENTREVISTA

Datos generales del informante sustituto (proxy) y/o cuidador(a)

ENTREVISTADOR: CONTINÚE LA ENTREVISTA HACIENDO TODAS LAS PREGUNTAS SOBRE EL ADULTO SELECCIONADO AL INFORMANTE SUSTITUTO/CUIDADOR(A)

**14a.11 ¿Qué edad tiene (en años) el cuidador(a)?** a14a11	Edad [___|___]
**14a.12 Sexo del cuidador(a)** a14a12	Hombre................................1<br>Mujer................................2

FILTRO: PERSONAS DE 20 A 59 AÑOS CON CÓDIGO 1 Ó 2 EN 14.0, PASE A FILTRO ANTES DE 0.1

XIV B. AUDICIÓN

Sr.(a), ahora queremos preguntarle respecto a su audición, es decir, la manera en que escucha.

Por favor dígame si…

**14b.01 ¿Normalmente (USTED/NOMBRE PERSONA MAYOR) usa audífono o aparato auditivo?** a14b01	Sí................................1<br>No................................2<br>NS/NR................................9
**14b.02 ¿Cómo es su alcance de oído/audición (con aparato)?** a14b02<br><br>ENTREVISTADOR: LA PREGUNTA SE HACE, USE O NO EL APARATO	Excelente................................1<br>Muy buena................................2<br>Buena................................3<br>Regular................................4<br>Mala................................5<br>No escucha (sordo)................................6<br>NS/NR................................9

XIV G. VISIÓN

Sr.(a), ahora queremos preguntarle respecto a su visión, es decir, la manera en que ve.

Por favor dígame si...

**14g.1 ¿Normalmente (USTED/NOMBRE PERSONA MAYOR) usa lentes?** a14g01	Sí................................1<br>No................................2<br>NS/NR................................9
**14g.2 ¿Cómo es su visión (con lentes)? ¿Es…?** a14g02<br><br>ENTREVISTADOR: LA PREGUNTA SE HACE, USE O NO LENTES	Excelente................................1<br>Muy buena................................2<br>Buena................................3<br>Regular................................4<br>Mala................................5<br>No ve (ciego)................................6<br>NS/NR................................9

XIV C. AUTOESTIMA Y SATISFACCIÓN

FILTRO: SI EL INFORMANTE SUSTITUTO (PROXY) Y/O CUIDADOR ES QUIEN CONTESTA EL CUESTIONARIO, PASE A 14e.01

Ahora nos gustaría hacerle algunas preguntas de opinión en relación a la vida en general y sobre su vida actual.

14c.01 ¿Se considera usted una persona valiosa? a14c01

NO LEA LAS OPCIONES Y ANOTE LA RESPUESTA

Sí……………………………………………………......................1
No………………………………………………….......................2
A veces (espontánea)…......……………….........................3
NS/NR……………………………..…...................................9

Por favor díganos qué tan satisfecho(a) está con los siguientes aspectos.

14c.02

¿Qué tan satisfecho(a) está …

LEA LAS OPCIONES Y ANOTE SÓLO UNA RESPUESTA

		Muy satisfecho(a)	satisfecho(a)	insatisfecho(a)	Muy insatisfecho(a)	No aplica	NS/NR	Código
a)	con su salud? a14c021	1	2	3	4		9	[____]
b)	consigo mismo(a) (con usted mismo(a))? a14c022	1	2	3	4		9	[____]
c)	con su capacidad para realizar sus actividades de la vida diaria (cotidianas)? a14c023	1	2	3	4		9	[____]
d)	con sus relaciones personales? a14c024	1	2	3	4		9	[____]
e)	con las condiciones del lugar en donde vive? a14c025	1	2	3	4		9	[____]
f)	con la relación que tiene con sus hijos? a14c026	1	2	3	4	5	9	[____]
g)	En general, ¿qué tan satisfecho(a) está con su vida considerando todas las cosas que le dije? a14c027	1	2	3	4		9	[____]

Ahora le voy hacer algunas preguntas que se relacionan con usted, su familia y su hogar.

14c.03 ¿Considera usted que su familia lo(la) toma en cuenta para las decisiones del gasto del hogar? a14c03

Sí……………………………………………………......................1
No………………………………………………….......................2
No aplica...................…......…………….........................3
NS/NR……………………………..…...................................9

14c.04 ¿Considera usted que su familia lo(la) toma en cuenta para las decisiones importantes del hogar? a14c04

Sí……………………………………………………......................1
No………………………………………………….......................2
No aplica...................…......…………….........................3
NS/NR……………………………..…...................................9

14c.05 ¿Aporta usted para los gastos del hogar? a14c05

Sí……………………………………………………......................1
No………………………………………………….......................2
No aplica...................…......…………….........................3
NS/NR……………………………..…...................................9

XIV E. FUNCIONALIDAD

ACTIVIDADES BÁSICAS DE LA VIDA DIARIA

Ahora quisiera preguntarle sobre DIFICULTADES que DEBIDO A UN PROBLEMA DE SALUD algunas personas tienen para realizar ciertas actividades importantes para la vida diaria. Por favor dígame si tiene alguna dificultad con cada una de las actividades que le voy a mencionar. Si usted. no hace ninguna de las siguientes actividades (por ejemplo, porque no lo acostumbra), simplemente dígamelo.

No incluya dificultades que cree que durarán menos de tres meses.

**14e.01 Debido a un problema de salud, ¿(USTED/NOMBRE PERSONA MAYOR) tiene dificultad para cruzar de un cuarto a otro caminando?** a14e01<br>LEA LAS OPCIONES Y ANOTE LA RESPUESTA	Sí……………………………………………………1<br>No……………………………………………………2<br>No puede………………………………………………3<br>No lo hace………………………………………………4<br>NS/NR……………………………………………………9
**14e.02 Debido a un problema de salud, ¿(USTED/NOMBRE PERSONA MAYOR) tiene dificultad para bañarse en una tina o regadera (incluyendo entrar y salir del baño)?** a14e02<br>LEA LAS OPCIONES Y ANOTE LA RESPUESTA	Sí……………………………………………………1<br>No……………………………………………………2<br>No puede………………………………………………3<br>No lo hace………………………………………………4<br>NS/NR……………………………………………………9
**14e.03 Debido a un problema de salud, ¿(USTED/NOMBRE PERSONA MAYOR) tiene dificultad al acostarse y levantarse de la cama?** a14e03<br>LEA LAS OPCIONES Y ANOTE LA RESPUESTA	Sí……………………………………………………1<br>No……………………………………………………2<br>No puede………………………………………………3<br>No lo hace………………………………………………4<br>NS/NR……………………………………………………9
**14e.04 Debido a un problema de salud, ¿(USTED/NOMBRE PERSONA MAYOR) tiene dificultad para vestirse (incluyendo ponerse los zapatos y los calcetines o medias)?** a14e04<br>LEA LAS OPCIONES Y ANOTE LA RESPUESTA	Sí……………………………………………………1<br>No……………………………………………………2<br>No puede………………………………………………3<br>No lo hace………………………………………………4<br>NS/NR……………………………………………………9
ENTREVISTADOR: SI EL ENTREVISTADO CONTESTÓ QUE SÍ EN POR LO MENOS UNA DE LAS ACTIVIDADES MENCIONADAS ANTERIORMENTE, PREGUNTE:<br>**14e.05 ¿Alguien le ayuda para realizar esta(s) actividad(es)?** a14e05	Sí……………………………………………………1<br>No……………………………………………………2<br>NS/NR……………………………………………………9

XIV E. FUNCIONALIDAD

ACTIVIDADES INSTRUMENTALES DE LA VIDA DIARIA

Ahora quisiera preguntarle sobre DIFICULTADES que DEBIDO A UN PROBLEMA DE SALUD algunas personas tienen para realizar ciertas actividades importantes para la vida diaria. Por favor dígame si tiene alguna dificultad con cada una de las actividades que le voy a mencionar. Si usted no hace ninguna de las siguientes actividades (por ejemplo, porque no lo acostumbra), simplemente dígamelo. No incluya dificultades que cree que durarán menos de tres meses.

Voy a mencionarle algunas actividades comunes de la vida diaria. Por favor dígame si DEBIDO A UN PROBLEMA DE SALUD usted tiene alguna dificultad para realizarlas.

Pregunta	Respuestas
**14e.06 Debido a un problema de salud, ¿(USTED/NOMBRE PERSONA MAYOR) tiene dificultad en preparar una comida caliente?** a14e06<br>LEA LAS OPCIONES Y ANOTE LA RESPUESTA	Sí.....1<br>No.....2<br>No puede.....3<br>No lo hace.....4<br>NS/NR.....9
**14e.07 Debido a un problema de salud, ¿(USTED/NOMBRE PERSONA MAYOR) tiene dificultad en hacer compras de víveres o ir al mercado?** a14e07<br>LEA LAS OPCIONES Y ANOTE LA RESPUESTA	Sí.....1<br>No.....2<br>No puede.....3<br>No lo hace.....4<br>NS/NR.....9
**14e.08 Debido a un problema de salud, ¿(USTED/NOMBRE PERSONA MAYOR) tiene dificultad en tomar sus medicamentos si toma alguno o tuviera que tomar alguno?** a14e08<br>LEA LAS OPCIONES Y ANOTE LA RESPUESTA	Sí.....1<br>No.....2<br>No puede.....3<br>No lo hace.....4<br>NS/NR.....9
**14e.09 Debido a un problema de salud, ¿(USTED/NOMBRE PERSONA MAYOR) tiene dificultad para manejar su dinero?** a14e09<br>LEA LAS OPCIONES Y ANOTE LA RESPUESTA	Sí.....1<br>No.....2<br>No puede.....3<br>No lo hace.....4<br>NS/NR.....9
ENTREVISTADOR: SI EL ENTREVISTADO CONTESTÓ QUE SÍ EN POR LO MENOS UNA DE LAS ACTIVIDADES MENCIONADAS ANTERIORMENTE, PREGUNTE:<br>**14e.10 ¿Alguien le ayuda para realizar esta(s) actividad(es)?** a14e10	Sí.....1<br>No.....2<br>NS/NR.....9
**14e.11 En los últimos 3 meses, ¿ha comido menos por falta de apetito, problemas digestivos, dificultades de masticación o deglución?** a14e11<br>LEA LAS OPCIONES Y ANOTE LA RESPUESTA	Ha comido mucho menos.....1<br>Ha comido menos.....2<br>Ha comido igual.....3<br>NS/NR.....9

XIV F. CAÍDAS

Pregunta	Respuestas
**14f.02 ¿Cuántas veces (USTED/NOMBRE PERSONA MAYOR) se ha caído en los últimos doce meses?** a14f02	Número de veces [____]____]<br>Ninguna.....00→ PASE A FILTRO ANTES DE 0.1<br>NS/NR.....99
**14f.03 ¿Cuántas veces ha recibido atención médica a consecuencia de esas caídas?** a14f03	Número de veces [____]____]<br>NS/NR.....99

Individual_Adultos

FILTRO: SI EL INFORMANTE SUSTITUTO (PROXY) Y/O CUIDADOR ES QUIEN RESPONDE, PASE A 2.01h Y CONTINÚE CON EL CUESTIONARIO

**0.1 En el último año, ¿le han dado alguna información sobre las consecuencias por el uso de tabaco, alcohol y otras drogas?** a001	Sí...1<br>No..2 → PASE A 1.01
**0.2 ¿Considera que esta información es útil para prevenir las consecuencias por el uso de tabaco, alcohol y otras drogas?** a002	Sí...1<br>No..2<br>NS/NR..9

I. SOBREPESO Y OBESIDAD

SILUETAS DE STUNKARD

MUESTRE LAS FIGURAS SEGÚN SEA EL SEXO DEL ENTREVISTADO

A continuación, le voy ha mostrar unas figuras corporales.

1.01 ¿Qué silueta corporal considera que se parece a usted en este momento?

1.01a ¿En cuál de las siluetas corporales preferiría estar usted?

NS/NR…………………………….….................................9

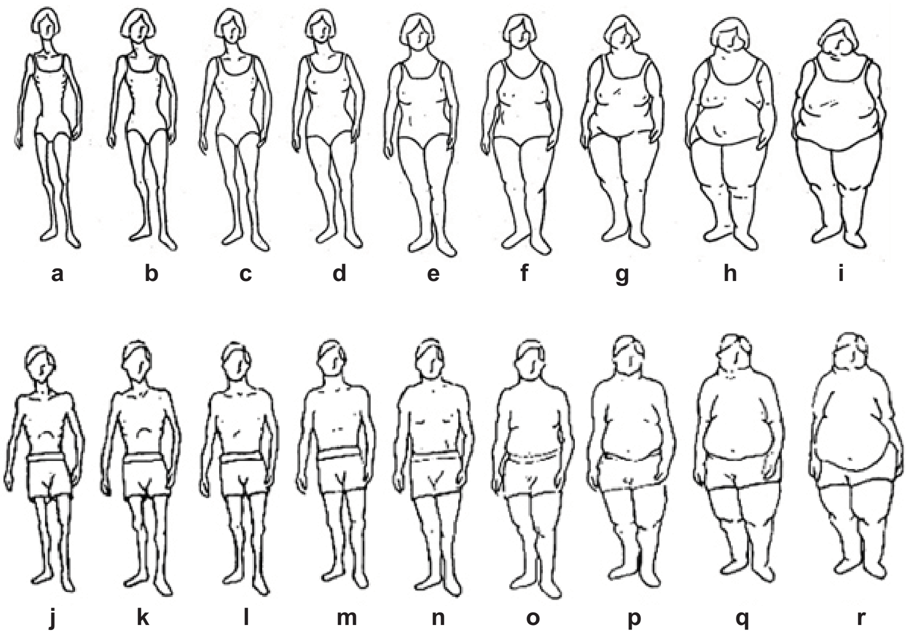

1.01 [______] a101m a101h

1.01a [______] a101am a101ah

II. DEPRESIÓN

A continuación le mencionaré sentimientos o conductas que usted haya podido experimentar. Por favor, dígame con qué frecuencia se ha sentido así durante la ÚLTIMA SEMANA.

2.01

Durante la última semana...

ENTREVISTADOR: LEA LAS OPCIONES DE RESPUESTA PARA CADA PREGUNTA Y ANOTE SÓLO UNA

		Rara vez o nunca (menos de un día)	Pocas veces o alguna vez (1 - 2 días)	Un número de veces considerable (3 - 4 días)	Todo el tiempo o la mayoría del tiempo (5 - 7 días)
a)	¿sentía como si no pudiera quitarse de encima la tristeza?	a201_a 1	2	3	4
b)	¿le costaba concentrarse en lo que estaba haciendo?	a201_b 1	2	3	4
c)	¿se sintió deprimido/a?	a201_c 1	2	3	4
d)	¿le parecía que todo lo que hacía era un esfuerzo?	a201_d 1	2	3	4
e)	¿no durmió bien?	a201_e 1	2	3	4
f)	¿disfrutó de la vida?	a201_f 1	2	3	4
g)	¿se sintió triste?	a201_g 1	2	3	4 → CUALQUIER OPCIÓN PASE A 2.02

Pregunta	Respuesta
**2.01h ¿Durante la última semana (NOMBRE PERSONA MAYOR) actuó como si estuviera triste, con el ánimo bajo?** a201_h	Sí....1<br>No....2<br>No sabe....8<br>No responde....9<br>(2, 8, 9) → PASE A 2.02
**2.01i ¿Qué tanto afecta esto a (NOMBRE PERSONA MAYOR)?** a201_i	Ligero (notable, pero no cambios significativos)....1<br>Moderado (significativo, pero no cambios dramáticos).....2<br>Severo (muy marcado y prominente, cambio dramático)...3<br>NS/NR....9
**2.02 ¿Alguna vez le ha dicho un médico u otro personal de salud que sufre o ha sufrido depresión?** a202	Sí....1<br>No....2 → PASE A 3.01
**2.03 ¿Le mandaron medicinas o algún otro tipo de tratamiento para la depresión?** a203	Sí....1<br>No....2<br>NS/NR....9
**2.04 Durante las últimas 2 semanas, ¿ha tomado algún medicamento o ha seguido con algún otro tipo de tratamiento para la depresión?** a204	Sí....1<br>No....2<br>NS/NR....9

III. DIABETES MELLITUS

Pregunta	Respuestas
**3.01 ¿Algún médico le ha dicho que tiene diabetes o el azúcar alta en la sangre?** a301	Sí.....1<br>No.....2 → PASE A 4.01
**3.02 ¿Hace cuánto tiempo le dijo su médico por primera vez que tenía diabetes o el azúcar alta en la sangre?**	Meses [___|___] a302a Años [___|___] a302b<br>Menos de un mes.....0000<br>NS/NR.....9999

FILTRO: SI ES MUJER CONTINÚE, SI ES HOMBRE PASE A LA PREGUNTA 3.05

Pregunta	Respuestas
**3.03 ¿Algún médico le ha diagnosticado diabetes durante alguno de sus embarazos?** a303	Sí.....1<br>No.....2<br>Nunca he estado embarazada.....3
**3.05 En los últimos 12 meses, ¿cuántas veces acudió al médico para controlar su diabetes (sin contar las visitas al servicio de urgencias?** a305	Veces [___|___]<br>Ninguna.....00 → PASE A 3.07<br>NS/NR.....99 → PASE A 3.07
**3.06 ¿En qué institución se atiende para controlar su diabetes?**<br>PUEDE ANOTAR MÁS DE UNA OPCIÓN	Seguro Social (IMSS).....01 a306a<br>ISSSTE.....02 a306b<br>ISSSTE Estatal.....03 a306c<br>Pemex.....04 a306d<br>Defensa.....05 a306e<br>Marina.....06 a306f<br>Centro de Salud u Hospital de la SSA.....07 a306g<br>IMSS Oportunidades.....08 a306h<br>Consultorios dependientes de farmacias.....09 a306i<br>Médico privado.....10 a306j<br>Otro lugar (especifique)____ a306esp ____77 a306k<br>NS/NR.....99 a306l
**3.07 ¿Actualmente toma pastillas o le aplican insulina para controlar su azúcar?** a307	**Sí, ¿cuál?**<br>Solo insulina.....1<br>Solo pastillas.....2 → PASE A 3.09<br>Ambas.....3<br>Ninguno.....4 → PASE A 3.09

III. DIABETES MELLITUS

Pregunta	Respuestas
**3.08 ¿Cuántas veces y con que frecuencia se aplica la insulina?**	Veces [___|___] Frecuencia [_____]<br>Diario.................. a308b .... a308a ....1<br>A la semana..2<br>NS/NR...999
**3.09 ¿Actualmente lleva algún otro tratamiento para controlar su azúcar?**<br><br>PUEDE ANOTAR MÁS DE UNA OPCIÓN	**Sí, ¿cuál?**<br>Plan de alimentación (dieta)..............................1 a309a<br>Realiza algún plan de ejercicio físico.........................2 a309b<br>Homeopatía (chochos)...3 a309c<br>Herbolaria..4 a309d<br>Medicina alternativa...5 a309e<br>No...8 a309f
**3.10 Durante los últimos 12 meses, ¿qué exámenes le hizo u ordenó su médico para vigilar su azúcar?**<br><br>PUEDE ANOTAR MÁS DE UNA OPCIÓN	Tiras reactivas en orina.............................. a310a . 1<br>Tiras reactivas en sangre............................. a310b ..2<br>Examen general de orina.............................. a310c ..3<br>Determinación de glucosa en sangre venosa... a310d . 4<br>Determinación de hemoglobina glucosilada..... a310e ..5<br>Examen para medir el nivel de proteínas en la orina (microalbuminuria).. a310f ..6<br>Automonitoreo.. a310g ..7<br>Ninguno.. a310h ..8 → PASE A 3.12
**3.11 Durante los últimos 12 meses, ¿cuántas veces se realizó esta prueba?**<br><br>PREGUNTE PARA CADA UNO DE LOS EXÁMENES QUE LE RESPONDIÓ EN LA PREGUNTA 3.10	Examen 1 Veces [___|___] a311e1<br>Examen 2 Veces [___|___] a311e2<br>Examen 3 Veces [___|___] a311e3<br>Examen 4 Veces [___|___] NS/NR......99 a311e4<br>Examen 5 Veces [___|___] a311e5<br>Examen 6 Veces [___|___] a311e6<br>Examen 7 Veces [___|___] a311e7
**3.12 Debido a la diabetes, durante los últimos 12 meses ¿qué medidas preventivas ha seguido para evitar complicaciones?**<br><br>PUEDE ANOTAR MÁS DE UNA OPCIÓN	Revisión oftalmológica (no lentes).............................1 a312a<br>Toma una aspirina diario...2 a312b<br>Revisión de pies...3 a312c<br>Examen general de orina y micro albuminuria.................4 a312d<br>No realiza ninguna medida preventiva..........................5 a312e<br>Otro (especifique)_________ a312esp _________7 a312f
**3.13 ¿Debido a la diabetes …**<br><br>LEA LAS OPCIONES Y CIRCULE SÓLO UN CÓDIGO PARA CADA OPCIÓN	Sí No<br>ha tenido ulceras en piernas o pies que tarden en sanar más de 4 semanas?.. 1 2 a313a<br>le han amputado alguna parte del cuerpo?............... 1 2 a313b<br>le ha disminuido su visión?....................................... 1 2 a313c<br>ha sufrido daño en la retina?.................................... 1 2 a313d<br>ha perdido la vista?... 1 2 a313e<br>le han hecho diálisis?.. 1 2 a313f<br>ha sufrido de un infarto?... 1 2 a313g<br>sufrió de un coma diabético?.................................... 1 2 a313h<br>sufre ardor, dolor o pérdida de la sensibilidad en la planta de los pies?.. 1 2 a313i

IV. HIPERTENSIÓN ARTERIAL

Pregunta	Respuestas
**4.01 ¿Algún médico le ha dicho que tiene la presión alta o hipertensión?** a401	Sí.....1<br>No.....2 → PASE A 5.01
**4.02 ¿Hace cuánto tiempo le dijo su médico que tiene la presión alta o hipertensión?**	Meses [___|___] a402a Años [___|___] a402b<br>Menos de un mes.....0000<br>NS/NR.....9999

FILTRO: SI ES MUJER CONTINÚE, SI ES HOMBRE PASE A LA PREGUNTA 4.05

Pregunta	Respuestas
**4.03 ¿Algún médico le diagnosticó presión alta durante alguno de sus embarazos?** a403	Sí.....1<br>No.....2<br>Nunca he estado embarazada.....3<br>(2, 3) → PASE A 4.05
**4.04 ¿Algún médico o personal de salud le diagnosticó preeclampsia solamente durante el tiempo en que estuvo embarazada?** a404	Sí.....1 → PASE A 4.08<br>No.....2
**4.05 ¿Actualmente toma alguna medicina (pastillas) para controlar su presión alta?** a405	Sí.....1<br>No.....2<br>NS/NR.....9<br>(2, 9) → PASE A 4.08
**4.06 ¿En que institución se atiende para controlar su presión alta?**<br>PUEDE ANOTAR MÁS DE UNA OPCIÓN	Seguro Social (IMSS).....01 a406a<br>ISSSTE.....02 a406b<br>ISSSTE Estatal.....03 a406c<br>Pemex.....04 a406d<br>Defensa.....05 a406e<br>Marina.....06 a406f<br>Centro de Salud u Hospital de la SSA.....07 a406g<br>IMSS Oportunidades.....08 a406h<br>Consultorios dependientes de farmacias.....09 a406i<br>Médico privado.....10 a406j<br>Otro lugar (especifique)______ a406esp ______77 a406k<br>NS/NR.....99 a406l
**4.07 ¿Actualmente lleva otro tratamiento para controlar su presión alta?**<br>PUEDE ANOTAR MÁS DE UNA OPCIÓN	**Sí, ¿cuál?**<br>Plan de alimentación (dieta).....1 a407a<br>Realiza algún plan de ejercicio físico.....2 a407b<br>Homeopatía (chochos).....3 a407c<br>Herbolaria.....4 a407d<br>Disminución en el consumo de sal.....5 a407e<br>Medicina alternativa.....6 a407f<br>No.....7 a407g
**4.08 En los últimos 12 meses, ¿cuántas veces se tomó o le tomaron la presión?**	Veces [___|___] a408b Frecuencia [____] a408a<br>NS/NR.....999<br>**Frecuencia:**<br>Diario.....1<br>Semanal.....2<br>Mensual.....3

V. ENFERMEDAD CARDIOVASCULAR

5.01 ¿Ha tenido alguna vez un dolor fuerte en el pecho, con falta de aire o gran malestar que durara media hora o más? a501

Sí...1
No...2

5.02 ¿Le ha dicho el médico que usted tiene o tuvo...

	Sí	No	
un infarto?...	1	2	a502a
angina de pecho?..	1	2	a502b
insuficiencia cardiaca?..	1	2	a502c
otra enfermedad del corazón? (especifique)______	1	2	a502d a502desp

VI. HIPERCOLESTEROLEMIA Y ENFERMEDAD NEUROVASCULAR

6.01 ¿Alguna vez le han medido el colesterol en la sangre? a601

Sí y lo encontraron normal...1 → PASE A 6.03
Sí y lo encontraron alto...2
No..3 ⎤
NS/NR...9 ⎦ → PASE A 6.03

6.02 ¿En los últimos 12 meses, recibió algún tratamiento para el colesterol alto?

PUEDE ANOTAR MÁS DE UNA OPCIÓN

Sí, ¿cuál?
Pravastatina, simvastatina, atorvastatina, rosuvastatina o fluvastatina...1 a602a
Disminuir la ingesta de grasa o colesterol en los alimentos..2 a602b
Aumento de actividad física......................................3 a602c
No...4 a602d

6.03 ¿Alguna vez le han medido los triglicéridos en la sangre? a603

Sí y los encontraron normales......................................1
Sí y los encontraron altos..2
No...3
NS/NR..9

6.04 ¿Alguna vez le ha dicho su médico que tuvo una embolia o un infarto cerebral? a604

Sí...1
No...2
NS/NR..9

FILTRO: SI EL INFORMANTE SUSTITUTO (PROXY) Y/O CUIDADOR ES QUIEN RESPONDE, PASE A 9.15 Y CONTINÚE CON EL CUESTIONARIO

VII. ANTECEDENTES HEREDO FAMILIARES

Ahora le preguntaré sobre algunas enfermedades que pudieran haber tenido sus padres.

FAMILIAR(ES)	7.01 ¿Su padre o madre tiene o tuvo diabetes o azúcar alta en la sangre? LEA TODAS LAS OPCIONES	7.02 ¿A qué edad le diagnosticaron a su (PADRE O MADRE) diabetes o azúcar alta en la sangre?	7.03 ¿Su padre o madre tiene o tuvo hipertensión o presión alta? LEA TODAS LAS OPCIONES	7.04 ¿A qué edad le diagnosticaron a su (PADRE O MADRE) la presión alta?	7.05 ¿Su padre o madre tuvo un infarto? LEA TODAS LAS OPCIONES	7.06 ¿A qué edad su (PADRE O MADRE) tuvo su primer infarto?
	Sí No NS/NR	Edad	Sí No NS/NR	Edad	Sí No NS/NR	Edad
a) Padre....................	a701a<br>1 2 9<br>(2, 9) PASE A 7.03	a702a<br>[___|___]<br>NS/NR.............99	a703a<br>1 2 9<br>(2, 9) PASE A 7.05	a704a<br>[___|___]<br>NS/NR.............99	a705a<br>1 2 9<br>(2, 9) PASE AL INCISO "b"	a706a<br>[___|___]<br>NS/NR.............99
b) Madre...................	a701b<br>1 2 9<br>(2, 9) PASE A 7.03	a702b<br>[___|___]<br>NS/NR.............99	a703b<br>1 2 9<br>(2, 9) PASE A 7.05	a704b<br>[___|___]<br>NS/NR.............99	a705b<br>1 2 9<br>(2, 9) PASE A FILTRO ANTES DE 8.01	a706b<br>[___|___]<br>NS/NR.............99

Individual_Adultos

VIII. SALUD REPRODUCTIVA

FILTRO: APLICAR SÓLO A MUJERES Y HOMBRES DE 20 A 49 AÑOS

PERSONAS DE 50 AÑOS Y MÁS PASE A FILTRO ANTES DE 9.01

8.01 ¿A qué edad tuvo su primera relación sexual? a801

Edad [___|___]
No ha tenido relaciones sexuales....00 → PASE A FILTRO ANTES DE 8.07
No recuerda....88
NS/NR....99

8.02 La primera vez que tuvo relaciones sexuales, ¿qué hicieron o qué usaron usted o su pareja para evitar un embarazo o una infección de transmisión sexual?

PUEDE ANOTAR MÁS DE UNA OPCIÓN

Condón o preservativo masculino....01 a802a
Pastillas de anticoncepción de emergencia o del día siguiente....02 a802b
Pastillas o píldoras....03 a802c
Inyecciones....04 a802d
Dispositivo, DIU o aparato....05 a802e
Implantes, tubos o norplant....06 a802f
Óvulos, jaleas, espuma o diafragma....07 a802g
Operación femenina, OTB o ligadura de trompas....08 a802h
Operación masculina o vasectomía....09 a802i
Ritmo, calendario, abstinencia periódica, termómetro, billings....10 a802j
Retiro o coito interrumpido....11 a802k
Otro (especifique) a802esp ____77 a802l
Nada....13 a802m
No recuerda....88 a802n
NS/NR....99 a802o

8.03 En su última relación sexual, ¿qué hicieron usted o su pareja para evitar un embarazo o una infección de transmisión sexual?

PUEDE ANOTAR MÁS DE UNA OPCIÓN

a803a a803b a803c a802d a803e a803f a803g a803h a803i a803j a803k a803l a803m a803n a803o

Condón o preservativo masculino....01 → PASE A 8.02b
Pastillas de anticoncepción de emergencia o del día siguiente....02
Pastillas o píldoras....03
Inyecciones....04
Dispositivo, DIU o aparato....05
Implantes, tubos o norplant....06
Óvulos, jaleas, espuma o diafragma....07
Operación femenina, OTB o ligadura de trompas....08
Operación masculina o vasectomía....09
Ritmo, calendario, abstinencia periódica, termómetro, billings....10
Retiro o coito interrumpido....11
Otro (especifique) a803esp ____77
Nada....12 → PASE A FILTRO ANTES DE 8.07
No recuerda....88
NS/NR....99

8.04 ¿Y además utilizaron condón masculino? a804

Sí....1
No....2 → PASE A FILTRO ANTES DE 8.07
NS/NR....9

8.02b ¿Por qué usaron condón?

PUEDE ANOTAR MÁS DE UNA OPCIÓN

Para prevenir infección por VIH....1 a802_ba
Para prevenir una infección de transmisión sexual diferente al VIH....2 a802_bb
Para prevenir un embarazo no deseado....3 a802_bc
Porque me lo pidió mi pareja aunque desconozco el motivo....4 a802_bd
Otro (especifique) a802_bsp ____7 a802_be
NS/NR....9 a802_bf

VIII. SALUD REPRODUCTIVA

FILTRO: SI ES HOMBRES PASE A 8.33b, SI ES MUJER CONTINÚE

8.07 ¿Hace cuánto tiempo fue su última regla o menstruación?

Meses [___|___] a807a Años [___|___] a807b

Menos de un mes..96 → PASE A 8.11

Actualmente está reglando...97 → PASE A 8.11

No recuerda...88

NS/NR………………………………..…................................99

FILTRO: SI LA ÚLTIMA MENSTRUACIÓN FUE HACE MENOS DE TRES MESES PASE A 8.11, SI FUE HACE TRES MESES O MAS CONTINÚE

8.08 ¿Cuál fue la razón por la que usted dejó de reglar? a808

Está amamantando o en puerperio...............................01 → PASE A 8.12

Actualmente está embarazada.......................................02 → PASE A 8.12

Por menopausia natural...03

Le quitaron la matriz o los ovarios.................................04

Por quistes..05

Recibió radiaciones en la pelvis....................................06

Tomó medicamentos o quimioterapia............................07

Otras razones (especifique)__________ a808esp __________77

NS/NR………………………………..…................................99

8.11 ¿Alguna vez ha estado embarazada? a811

Sí...1

No...2 → PASE A 8.33b

NS/NR………………………………..…................................9 → PASE A 8.33b

8.11a ¿Está actualmente embarazada? a811a

Sí...1

No...2

NS/NR…...9

8.12 ¿Cuántos embarazos en total ha tenido? a812

NO OMITA EL EMBARAZO ACTUAL AUNQUE ESTE SEA EL PRIMERO

Número de embarazos [___|___]

No recuerda..88

NS/NR………………………………..…................................99

FILTRO: SI EN LA PREGUNTA 8.11a EL CÓDIGO ES 1 Y EN LA PREGUNTA 8.12 CÓDIGO ES 1 ó SI EN LA PREGUNTA 8.08 EL CÓDIGO ES 2 Y EN LA PREGUNTA 8.12 EL CÓDIGO ES 1 (EL EMBARAZO ACTUAL ES EL PRIMER EMBARAZO) PASE A 8.33b

8.13 ¿De estos embarazos cuantos han sido…

nacidos vivos, que no hayan fallecido?.........[_____|_____] a813_1

nacidos vivos, que hayan fallecido antes de cumplir un año de edad?........................[_____|_____] a813_2

nacidos muertos?..[_____|_____] a813_3

abortos?..[_____|_____] a813_4

VIII. SALUD REPRODUCTIVA

FILTRO:

1.- SI CONTESTO A 8.13 AL MENOS UN HIJO NACIDO VIVO QUE NO HA FALLECIDO CONTINÚE
2.- SI CONTESTO A 8.13 CUALQUIERA DE LAS OTRAS OPCIONES PASE A 8.33b

Pregunta	Respuestas
**8.15 ¿En qué día, mes y año nació su último(a) hijo(a) nacido(a) vivo(a) y que sigue vivo(a)?** a815	Día [___|___] Mes [___|___] Año [___|___|___|___]<br>No recuerda……………………….....................88888888<br>NS/NR...99999999

FILTRO: APLICAR SOLO A MUJERES CUYO ÚLTIMO NACIDO VIVO FUE A PARTIR DEL 2006, SI NO PASE A 8.31

Pregunta	Respuestas
**8.17 En total ¿cuántas veces la revisaron durante su último embarazo?** a817	Veces [___|___]<br>Nunca la revisaron...00 → PASE A 8.23<br>NS/NR...99
**8.18 ¿Quién la revisó la mayoría de las veces durante su último embarazo?** a818	Médico……….............................………….................1<br>Enfermera(o)......................………...............................2<br>Promotor(a), auxiliar o asistente de salud.........................3<br>Partera profesional técnica..4<br>Partera tradicional o empírica..5<br>Otro personal de salud..6<br>No recuerda...8<br>NS/NR...9
**8.19 ¿En qué lugar la revisaron la mayoría de las veces durante este embarazo?** a819	Seguro Social (IMSS)...01<br>ISSSTE...02<br>ISSSTE Estatal...03<br>Pemex...04<br>Defensa..05<br>Marina...06<br>Centro de Salud u Hospital de la SSA...........................07<br>IMSS Oportunidades..08<br>Consultorios dependientes de farmacias.......................09<br>Consultorio, clínica u hospital privado............................10<br>Casa de la partera...11<br>Casa de la entrevistada..12<br>Otro lugar (especifique)___a819esp___77<br>NS/NR...99
**8.20 ¿Cuántos meses de embarazo tenía cuando la revisaron por primera vez?** a820	Meses [___|___]<br>No recuerda..88<br>NS/NR...99

VIII. SALUD REPRODUCTIVA

8.21 Durante su último embarazo cuando visitó al médico, enfermero(a) o algún otro personal de salud, ¿le realizó algo de lo siguiente al menos una vez?

LEA TODAS LAS OPCIONES Y CIRCULE SÓLO UN CÓDIGO PARA CADA OPCIÓN

	Sí	No	NS/NR	
¿La midieron?	1	2	9	a821a
¿La pesaron?	1	2	9	a821b
¿Le tomaron la presión arterial?	1	2	9	a821c
¿Le realizaron examen(es) general(es) de orina?	1	2	9	a821d
¿Le realizaron examen(es) de sangre?	1	2	9	a821e
¿Le midieron su nivel de azúcar en sangre?	1	2	9	a821f
¿Le realizaron la prueba de detección de la sífilis (VRDL)?	1	2	9	a821g
¿Le realizaron la prueba para detectar Virus de Inmunodeficiencia Humana (VIH)?	1	2	9	a821h
¿Le hicieron un ultrasonido?	1	2	9	a821i
¿La vacunaron contra el tétanos?	1	2	9	a821j
¿Le mandaron ácido fólico?	1	2	9	a821k
¿Le mandaron vitaminas, hierro o algún suplemento alimenticio?	1	2	9	a821l

8.22 Durante su último embarazo, ¿le diagnosticaron o le dijeron que tenía…

LEA TODAS LAS OPCIONES Y CIRCULE SÓLO UN CÓDIGO PARA CADA OPCIÓN

	Sí	No	NS/NR	
la presión alta?	1	2	9	a822a
sangrado vaginal?	1	2	9	a821b
amenaza de aborto?	1	2	9	a822c
preeclampsia o eclampsia?	1	2	9	a822d
azúcar alta en la sangre o diabetes?	1	2	9	a822e
anemia?	1	2	9	a822f
una infección urinaria?	1	2	9	a822g
una infección de transmisión sexual?	1	2	9	a822h
infección por VIH o SIDA?	1	2	9	a822i
alguna otra enfermedad o padecimiento?	1	2	9	a822j

8.23 ¿Quién la atendió en su último parto? a823

- Médico……1
- Enfermera……2
- Promotora, auxiliar o asistente de salud……3
- Partera profesional técnica……4
- Partera tradicional o empírica……5
- Otro personal de salud……6
- Nadie……7
- No recuerda……8
- NS/NR……9

8.24 ¿En dónde la atendieron de su último parto? a824

- Seguro Social (IMSS)……01
- ISSSTE……02
- ISSSTE Estatal……03
- Pemex……04
- Defensa……05
- Marina……06
- Centro de Salud u Hospital de la SSA……07
- IMSS Oportunidades……08
- Consultorios dependientes de farmacias……09
- Consultorio, clínica u hospital privado……10
- Casa de la partera……11
- Casa de la entrevistada……12
- Otro lugar (especifique) a824esp ______ 77
- NS/NR……99

VIII. SALUD REPRODUCTIVA

8.25 ¿Tuvo alguna complicación en su último parto? a825

Sí...1
No...2
NS/NR..9
(No, NS/NR → PASE A 8.31)

8.26 En su último parto ¿le dijeron que tenía…

LEA TODAS LAS OPCIONES Y CIRCULE SÓLO UN CÓDIGO PARA CADA OPCIÓN

	Sí	No	NS/NR	
preeclampsia/eclampsia?.........................	1	2	9	a826a
sangrado vaginal abundante o hemorragia?..	1	2	9	a826b
aborto?..	1	2	9	a826c
amenaza de aborto?................................	1	2	9	a826d
parto obstruido?.......................................	1	2	9	a826e
mala posición del bebé?..........................	1	2	9	a826f
parto prematuro?.....................................	1	2	9	a826g
complicaciones debido a una enfermedad que ya tenía? (como diabetes, presión alta, etc.)......................	1	2	9	a826h

8.31 ¿Su último parto fue… a831

LEA TODAS LAS OPCIONES Y ANOTAR SÓLO UNA OPCIÓN

normal (vaginal)?..1
cesárea por urgencia?...2
cesárea programada?...3
NS/NR...9

8.28a ¿Cuánto pesó al nacer su último(a) hijo(a)?

NOTA: ÚLTIMO(A) HIJO(A) NACIDO(A) VIVO(A) Y QUE SIGUE VIVO(A)

Kilos l_____l . gramos l____l____l____l a828a_1

No lo pesaron...0000
NS/NR...9999

ENTREVISTADOR: ANOTE LA OPCIÓN DONDE INDIQUE DONDE OBTUVO LA INFORMACIÓN l____l a828a_2

De la cartilla del niño o de otro documento.......................1
Del recuerdo de su mamá, cuidadora o informante.............2
NS/NR...9

8.40 Cuando nació su último(a) hijo(a), ¿era... a840

NOTA: ÚLTIMO(A) HIJO(A) NACIDO(A) VIVO(A) Y QUE SIGUE VIVO(A)

muy grande?...1
más grande de lo normal?...2
de tamaño normal?...3
más pequeño(a) de lo normal?.......................................4
muy pequeño?...5
NS/NR...9

8.13d ¿Cuántos meses amamantó a su último(a) hijo(a)?

NOTA: ÚLTIMO(A) HIJO(A) NACIDO(A) VIVO(A) Y QUE SIGUE VIVO(A)

ENTREVISTADOR: SI NUNCA LE DIÓ PECHO, PONER 000.

SI MENOS DE 1 MES, ANOTE DÍAS.

SI MÁS DE 1 MES, ANOTE MESES.

CUANDO MÁS DE 1 AÑO, CONVIERTA A MESES: (EJEMPLOS: 1AÑO=12 M; 2 AÑOS=24 M; 3 AÑOS=36 M; 4 AÑOS=48 M)

Tiempo [_____]_____] a813db - Período [_____] a813da

Nunca le dió pecho......000
NS/NR........................999
Aún le da pecho...........888

Período:
Días..............................1
Meses..........................2

VIII. SALUD REPRODUCTIVA

**8.32 Después del nacimiento de su último(a) hijo(a), ¿le proporcionaron un método anticonceptivo antes de salir del hospital o del lugar donde la atendieron?** a832	Sí....1<br>No....2 → PASE A 8.33b<br>No recuerda....8 → PASE A 8.33b<br>NS/NR....9 → PASE A 8.33b
**8.32a ¿Qué método anticonceptivo le proporcionaron?** a832a	Condón o preservativo masculino....01<br>Pastillas de anticoncepción de emergencia o del día siguiente....02<br>Pastillas o píldoras....03<br>Inyecciones....04<br>Dispositivo, DIU o aparato....05<br>Implantes, tubos o norplant....06<br>Óvulos, jaleas, espuma o diafragma....07<br>Operación femenina, OTB o ligadura de trompas....08<br>Operación masculina o vasectomía....09<br>Otro (especifique) a832aesp ____ 77<br>NS/NR....99
**8.33b En los últimos 12 meses, ¿ha recibido condones de forma gratuita?** a833b	Sí....1<br>No....2 → PASE A 8.33a<br>NS/NR....9 → PASE A 8.33a
**8.33c Aproximadamente, ¿cuántos condones le dieron gratuitamente en los últimos 12 meses?** a833c	Condones [___|___|___]<br>NS/NR....999
**8.33d ¿En dónde le dieron los condones gratuitos?**<br><br>PUEDE ANOTAR MAS DE UNA OPCIÓN	Seguro Social (IMSS)....01 a833da<br>ISSSTE....02 a833db<br>ISSSTE Estatal....03 a833dc<br>Pemex....04 a833dd<br>Defensa....05 a833de<br>Marina....06 a833df<br>Centro de Salud u Hospital de la SSA....07 a833dg<br>IMSS Oportunidades....08 a833dh<br>Consultorios dependientes de farmacias....09 a833di<br>Médico privado....10 a833dj<br>ONGs....11 a833dk<br>Servicio médico de escuelas (medio superior y universidad)....12 a833dl<br>Eventos masivos....13 a833dm<br>Ferias de la salud....14 a833dn<br>Establecimientos públicos....15 a833do<br>Otro lugar (especifique) a833desp ____ 77 a833dp<br>NS/NR....99 a833dq

VIII. SALUD REPRODUCTIVA

**8.33a En los últimos 12 meses, ¿ha recibido consulta médica para atenderse o recibir tratamiento para…**	Sí / No / NS/NR<br>verrugas genitales?.................................. 1 2 9 a833a_a<br>gonorrea?.. 1 2 9 a833a_b<br>sífilis?.. 1 2 9 a833a_c<br>Virus de Inmunodeficiencia Humana (VIH)? 1 2 9 a833a_d<br>otra infección de transmisión sexual? (especifique)________ a833aesp 1 2 9 a833a_e
**8.35 ¿Le han realizado alguna vez la prueba para detectar el virus de Inmunodeficiencia Humana (VIH)?** a835	Sí.......................................1<br>No......................................2<br>NS/NR...............................9<br>(2, 9) PASE A 8.37
**8.36 En los últimos 12 meses, ¿le han realizado una prueba para detectar Virus de Inmunodeficiencia Humana (VIH)?** a836	Sí.......................................1<br>No......................................2<br>NS/NR...............................9<br>(2, 9) PASE A 8.37
**8.35a ¿Conoce el resultado de la prueba?** a835a	Sí.......................................1<br>No......................................2<br>NS/NR...............................9
**8.37 Podría decirme, ¿cuál es el beneficio de tomar ácido fólico?** a837	Ayuda a prevenir algunas malformaciones al nacimiento (malformaciones del sistema nervioso central y de la columna)..........1<br>Otro (especifique)________ a837esp ________7<br>NS/NR...............................9

FILTRO: APLICAR SÓLO A MUJERES
HOMBRES PASE A FILTRO ANTES DE 8.34

**8.39 En los últimos 12 meses, ¿ha tomado ácido fólico?** a839	Sí.......................................1<br>No......................................2

FILTRO: APLICAR SÓLO A MUJERES Y HOMBRES DE 20 A 34 AÑOS QUE TIENEN RELACIONES SEXUALES
MUJERES DE 35 AÑOS Y MÁS PASE A FILTRO ANTES DE 8a.13a
HOMBRES Y MUJERES DE 20 A 34 AÑOS QUE NO HAN TENIDO RELACIONES SEXUALES PASE A FILTRO ANTES DE 9.01

**8.34 ¿Cuántas parejas sexuales ha tenido en su vida?** a834	Parejas [___|___]<br>No recuerda.........................88<br>NS/NR...............................99

VIIIA. NACIDOS VIVOS EN LOS ÚLTIMOS 5 AÑOS Y QUE MURIERON

FILTRO: SI ES MUJER DE 20 A 49 Y HA ESTADO EMBARAZADA CONTINÚE
SI ES HOMBRE, MUJER DE 50 Y MÁS, O MUJER DE 20 A 49 SIN ANTECEDENTE DE EMBARAZO, PASE A FILTRO ANTES DE 9.01

**8a.13a ¿Tuvo algún hijo(a) nacido vivo después de junio del año 2006 que haya muerto?** a8a13a	Sí....1<br>No....2 → PASE A FILTRO ANTES DE 9.01
**8a.13b ¿Cuál era el nombre de su último hijo(a) que murió?** a8a13b	____________ Nombre
**8a.13b_1 ¿Qué edad tenía (NOMBRE del hijo que murió) cuando murió?**<br>SI MENOS DE 1 MES, ANOTE DÍAS. SI MÁS DE 1 MES, ANOTE MESES.<br>ENTREVISTADOR: NO REDONDEE LOS MESES, REGISTRE MESES CUMPLIDOS.	Edad [____]____] a8a13b1b - Período [____] a8a13b1a<br>NS/NR....999<br>**Período:**<br>Días....1<br>Meses....2
**8a.13c ¿Cuánto pesó al nacer (NOMBRE del hijo que murió)?**<br>ÚLTIMO HIJO(A) QUE MURIÓ DESPUÉS DE JUNIO DEL 2006	Kilos I____I . gramos I____I____I____I a8a13c_1<br>No lo pesaron....0000<br>NS/NR....9999<br>ENTREVISTADOR: ANOTE LA OPCIÓN DONDE INDIQUE DONDE OBTUVO LA INFORMACIÓN I____I a8a13c_2<br>De la cartilla del niño o de otro documento....1<br>Del recuerdo de su mamá, cuidadora o informante....2<br>NS/NR....9
**8a.13d Cuando nació (NOMBRE del hijo que murió), ¿era..** a8a13d	muy grande?....1<br>más grande de lo normal?....2<br>de tamaño normal?....3<br>más pequeño(a) de lo normal?....4<br>muy pequeño?....5<br>NS/NR....9
**8a.13e ¿Cuántos meses amamantó a (NOMBRE del hijo que murió)?**<br>ENTREVISTADOR: ANOTE MESES CUMPLIDOS, NO REDONDEE LOS MESES.<br>SI MENOS DE 1 MES, ANOTE DÍAS. SI MÁS DE 1 MES, ANOTE MESES. CUANDO MÁS DE 1 AÑO, CONVIERTA A MESES:<br>(EJEMPLOS: 1AÑO=12 M; 2 AÑOS=24 M; 3 AÑOS=36 M; 4 AÑOS=48 M)	Tiempo [____]____] a8a13eb ------ Período [____] a8a13ea<br>Nunca le dió pecho...000<br>Toda su vida/ hasta que murió....888 → PASE A FILTRO ANTES DE 9.01<br>NS/NR....999<br>**Período:**<br>Días....1<br>Meses....2
**8a.13f ¿Cuál fue el motivo por el que nunca le dio pecho o se lo quitó a (NOMBRE del hijo que murió)?**<br>PUEDE ANOTAR MÁS DE UNA OPCIÓN	Me quedé sin leche....01 a8a13fa<br>Fue el mismo tiempo que amamantó al hijo anterior....02 a8a13fb<br>Tuve que salir a trabajar....03 a8a13fc<br>Me lo indicó el médico....04 a8a13fd<br>Me aconsejaron (familiar / amiga / vecina) que debía dejar....05 a8a13fe<br>Quedé embarazada....06 a8a13ff<br>Me enfermé....07 a8a13fg<br>Mi bebé se enfermó....08 a8a13fh<br>Mi bebé nació tan enfermo que no podía mamar....09 a8a13fi<br>Mi bebé no aumentaba de peso....10 a8a13fj<br>Mi bebé es adoptado/a....11 a8a13fk<br>Porque así lo preferí....12 a8a13fl<br>Otros (especifique)____ a8a13fes ____77 a8a13fm<br>NS/NR....99 a8a13fn

IX. VACUNACIÓN

FILTRO: ADULTOS DE 20 A 59 AÑOS CONTINÚE

ADULTOS DE 60 AÑOS O MÁS PASE A 9.15

Las siguientes preguntas se refieren a todas las vacunas que le han aplicado a partir de los 20 años, considere las vacunas que le aplicaron en la unidad médica, con el médico privado, durante campañas de vacunación o en su casa.

**9.01 ¿Me puede mostrar su Cartilla Nacional de Salud y/o el documento probatorio o comprobante en el que le registran las vacunas que le han aplicado?** a901a a901b<br>PUEDE ANOTAR HASTA DOS OPCIONES<br>NOTA: LAS POSIBLES COMBINACIONES EN CASO DE DAR DOS OPCIONES SON: 1 y 2, 2 y 3 ó 2 y 4	Sí, mostró la Cartilla Nacional de Salud (Hombres y Mujeres de 20 a 49 años)............................1<br>Sí, mostró documento probatorio o comprobante….........2<br>Si la tiene pero no la mostró..............…….….…..................3<br>No tiene cartilla...4<br>NS/NR...9
**9.05 ¿Le han aplicado la vacuna contra el sarampión y la rubéola (SR)?** a905	Sí, y está registrado en la Cartilla Nacional de Salud.......1<br>Sí, y está registrado en el documento probatorio o comprobante...2<br>Si, dice haberse aplicado la vacuna……….......................3<br>No………………………………………………….…..................4 } PASE A 9.09<br>NS/NR...9 } PASE A 9.09
**9.06 ¿Cuántas veces se la han aplicado?** a906	Veces [_____]<br>NS/NR...9
**9.09 ¿Le han aplicado la vacuna contra el tétanos?** a909	Sí, y está registrado en la Cartilla Nacional de Salud.......1<br>Sí, y está registrado en el documento probatorio o comprobante...2<br>Si, dice haberse aplicado la vacuna……….......................3<br>No………………………………………………….…..................4 } PASE A 10.A<br>NS/NR...9 } PASE A 10.A
**9.10 ¿Cuántas veces se la han aplicado?** a910	Veces [_____] } PASE A 10.A<br>NS/NR...9 } PASE A 10.A

IX. VACUNACIÓN

FILTRO: ADULTOS DE 60 AÑOS O MÁS CONTINÚE
ADULTOS DE 20 A 59 AÑOS PASE A 10.A

Las siguientes preguntas se refieren a todas las vacunas que le han aplicado a partir de los 60 años, considere las vacunas que le aplicaron en la unidad médica, con el médico privado, durante campañas de vacunación o en su casa.

9.15 ¿Me puede mostrar su Cartilla Nacional de Salud del Adulto Mayor y/o el documento probatorio o comprobante en el que le registran las vacunas que le han aplicado?

a915a
a915b

PUEDE ANOTAR HASTA DOS OPCIONES

NOTA: LAS POSIBLES COMBINACIONES EN CASO DE DAR DOS OPCIONES SON: 1 y 2, 2 y 3 ó 2 y 4

- Sí, mostró la Cartilla Nacional de Salud (Adultos mayores de 60 años o más)....1
- Sí, mostró documento probatorio o comprobante....2
- Si la tiene pero no la mostró....3
- No tiene cartilla....4
- NS/NR....9

FILTRO: ADULTOS DE 65 AÑOS CONTINÚE
ADULTOS DE 60 AÑOS A 64 AÑOS Y 11 MESES PASE A 9.24

9.20 ¿Le han aplicado la vacuna contra el Neumococo? a920

- Sí, y está registrado en la Cartilla Nacional de Salud....1
- Sí, y está registrado en el documento probatorio o comprobante....2
- Si, dice haberse aplicado la vacuna....3
- No....4
- NS/NR....9

9.24 ¿Le han aplicado la vacuna contra el tétanos? a924

- Sí, y está registrado en la Cartilla Nacional de Salud....1
- Sí, y está registrado en el documento probatorio o comprobante....2
- Si, dice haberse aplicado la vacuna....3
- No....4 → PASE A 9.27
- NS/NR....9 → PASE A 9.27

9.25 ¿Cuántas veces se la han aplicado la vacuna contra el tétanos? a925

Veces [_____]

NS/NR....9

9.27 A partir de septiembre del año pasado hasta el día de hoy, ¿le han aplicado la vacuna contra la influenza? a927

- Sí, y está registrado en la Cartilla Nacional de Salud....1
- Sí, y está registrado en el documento probatorio o comprobante....2
- Si, dice haberse aplicado la vacuna....3
- No....4
- NS/NR....9

X. PROGRAMAS PREVENTIVOS

FILTRO: PARA ADULTOS DE 20 AÑOS Y MÁS

10.A ¿Alguna vez le ha dicho un médico que tiene (tuvo) cáncer o un tumor maligno de cualquier tipo? a1000a

- Sí....1
- No....2 → PASE A 10.01
- NS/NR....9 → PASE A 10.01

10.B ¿Qué tipo de cáncer le dijeron que tenía? a1000a

- Piel (melanoma)....1
- Cérvix o cuello uterino....2
- Mama....3
- Próstata....4
- Estómago....5
- Leucemia/sangre....6
- Otro lugar (especifique)________________7

X. PROGRAMAS PREVENTIVOS

	10.01<br>**Durante los últimos 12 meses, ¿se ha realizado en alguna unidad de salud la ...**<br>Sí.............1<br>No............2<br>No sabe....9 → PASE A LA SIGUIENTE PRUEBA DE DETECCIÓN.		10.02<br>**¿En qué institución le dieron el servicio?**<br>Seguro Social (IMSS)............................01<br>ISSSTE..02<br>ISSSTE Estatal......................................03<br>Pemex...04<br>Defensa ....05<br>Marina..06<br>Centro de Salud u Hospital de la SSA....07<br>IMSS Oportunidades.............................08<br>Consultorios dependientes de farmacias...09<br>Médico privado......................................10<br>Otro lugar (especifique)_____________77<br>NS/NR...99
**a)**	**detección de tuberculosis? (análisis de flema o esputo)**	a1001a |_____|	|_____|_____| a1002a a1002asp
**b)**	**detección de sobrepeso u obesidad?**	a1001b |_____|	|_____|_____| a1002b a1002bsp
**c)**	**detección de colesterol o triglicéridos alto (examen de sangre)?**	a1001c |_____|	|_____|_____| a1002c a1002csp
	**ENTREVISTADOR: REVISE LA PREGUNTA 3.01, SI LA RESPUESTA ES SÍ (CÓDIGO 1) PASE AL SIGUIENTE PROGRAMA PREVENTIVO**		
**d)**	**detección de diabetes (prueba del azúcar)?**	a1001d |_____|	|_____|_____| a1002d a1002dsp
	**ENTREVISTADOR: REVISE LA PREGUNTA 4.01, SI LA RESPUESTA ES SÍ (CÓDIGO 1) PASE AL SIGUIENTE PROGRAMA PREVENTIVO**		
**e)**	**detección de hipertensión (toma de presión arterial)?**	a1001e |_____|	|_____|_____| a1002e a1002esp
	**LOS SIGUIENTES INCISOS SON SÓLO PARA HOMBRES, MUJERES PASE A 10.8**<br>**HOMBRES CON CÓDIGO 4 EN PREGUNTA 10.B PASAN A 11.01**		
**f)**	**detección de cáncer de próstata (análisis de sangre para detectar antígenos prostáticos)?**	a1001f |_____|	|_____|_____| a1002f a1002fsp
**g)**	**prueba de tacto rectal para detectar cáncer de próstata?**	a1001g |_____|	|_____|_____| a1002g a1002gsp
	**HOMBRES PASE A 11.01**		

X. PROGRAMAS PREVENTIVOS

		10.05<br>¿Cuál fue el resultado de su prueba?<br>Positivo..........1<br>Negativo.........2<br>No le entregaron el resultado.........3<br>No acudió por el resultado.........4<br>NS/NR............9<br>(2, 3, 4, 9 →) PASE A LA SIGUIENTE PRUEBA DE DETECCIÓN.	10.06<br>¿Recibió tratamiento?<br>Sí.............1<br>No............2<br>(1 →) PASE A LA SIGUIENTE PRUEBA DE DETECCIÓN.	10.07<br>¿Cuál fue la principal causa por la que no recibío tratamiento?<br>No requirió tratamiento.......01<br>No hay donde atenderse.....02<br>Es caro..............................03<br>No tenía dinero...................04<br>La unidad médica le queda lejos.........................05<br>Falta de confianza.............06<br>La tratan mal.....................07<br>No tuvo tiempo..................08<br>Fue pero no lo atendieron....09<br>Por pena............................10<br>No le explicaron lo que le ban hacer.......................11<br>Su esposo o pareja no le permite...........................12<br>En una prueba posterior no se encontró lesión.............13<br>Otro.....................................77<br>NS/NR.................................99<br>PASE A SIGUIENTE PRUEBA DE DETECCIÓN, ÚLTIMA PASE A 10.08
	→	→	→	→
a)	detección de tuberculosis? (análisis de flema o esputo)	a1005a |__|	a1006a |__|	a1007a |__|__|
b)	detección de sobrepeso u obesidad?	a1005b |__|	a1006b |__|	a1007b |__|__|
c)	detección de colesterol o triglicéridos alto (examen de sangre)?	a1005c |__|	a1006c |__|	a1007c |__|__|
ENTREVISTADOR: REVISE LA PREGUNTA 3.01, SI LA RESPUESTA ES SÍ (CÓDIGO 1) PASE AL SIGUIENTE PROGRAMA PREVENTIVO				
d)	detección de diabetes (prueba del azúcar)?	a1005d |__|	a1006d |__|	a1007d |__|__|
ENTREVISTADOR: REVISE LA PREGUNTA 4.01, SI LA RESPUESTA ES SÍ (CÓDIGO 1) PASE AL SIGUIENTE PROGRAMA PREVENTIVO				
e)	detección de hipertensión (toma de presión arterial)?	a1005e |__|	a1006e |__|	a1007e |__|__|
LOS SIGUIENTES INCISOS SON SÓLO PARA HOMBRES, MUJERES PASE A 10.8<br>HOMBRES CON CÓDIGO 4 EN PREGUNTA 10.B PASAN A 11.01				
f)	detección de cáncer de próstata (análisis de sangre para detectar antígenos prostáticos)?	a1005f |__|	a1006f |__|	a1007f |__|__|
g)	prueba de tacto rectal para detectar cáncer de próstata?	a1005g |__|	a1006g |__|	a1007g |__|__|
HOMBRES PASE A 11.01				

X. PROGRAMAS PREVENTIVOS

FILTRO: PARA MUJERES DE 20 Y MAS AÑOS

	10.08		10.09	10.10	10.11
	**Alguna vez en su vida, ¿un médico o enfermera, le ha realizado una...** Sí.............1 No............2 No sabe....9 → PASE A LA SIGUIENTE PRUEBA DE DETECCIÓN. →		**Durante el 2011, ¿acudió al módulo de medicina preventiva para…** Sí............................1 No............................2 →	**Durante el 2010, ¿acudió al módulo de medicina preventiva para …** Sí............................1 No............................2 →	**Durante el 2009, ¿acudió al módulo de medicina preventiva para …** Sí............................1 No............................2 →
**MUJERES CON CÓDIGO 2 EN PREGUNTA 10.B PASE A INCISO "c)"**					
**a)**	**prueba de Papanicolaou?** *Es cuando algún médico o enfermera con la ayuda de una espátula/hisopo/cepillo extrae una muestra de células del cérvix o del cuello de su matriz y la manda al laboratorio.*	|_____| a1008a	|_____| a1009a	|_____| a1010a	|_____| a1011a
**b)**	**Prueba del Papiloma Virus?** *El personal de salud o usted misma introduce un cepillo pequeño para frotar el cuello de su matriz/ útero y lo coloca en un tubo con un líquido especial. Este tubo se manda a un laboratorio.* MUESTRE LA TARJETA 1	|_____| a1008b	|_____| a1009b	|_____| a1010b	|_____| a1011b
**MUJERES CON CÓDIGO 3 EN PREGUNTA 10.B PASE A 11.01**					
**c)**	**Exploración clínica de los senos?** *El médico o enfermera palpa los senos, para identificar cambios de coloración o de temperatura, engrosamiento de la piel o la presencia de bolitas muy duras o dolorosas.*	|_____| a1008c	|_____| a1009c	|_____| a1010c	|_____| a1011c
**d)**	**Mastografía?** *Se trata de una radiografía de sus senos con un aparato como el de la tarjeta que le mostraré.* MUESTRE LA TARJETA 2	|_____| a1008d	|_____| a1009d	|_____| a1010d	|_____| a1011d

X. PROGRAMAS PREVENTIVOS

FILTRO: PARA MUJERES DE 20 Y MAS AÑOS

		10.12	10.13	10.14
		**¿Hace cuánto tiempo le hicieron su última (MENCIONE OPCIÓN DE 10.8)**<br><br>SI ES HACE 4 AÑOS O MÁS, PASE A LA SIGUIENTE PRUEBA DE DETECCIÓN<br><br>NS/NR.........9999<br>→	**¿En qué institución le dieron el servicio?**<br>Seguro Social (IMSS)............................01<br>ISSSTE...02<br>ISSSTE Estatal.....................................03<br>Pemex..04<br>Defensa ....05<br>Marina..06<br>Centro de Salud u Hospital de la SSA....07<br>IMSS Oportunidades.............................08<br>Consultorios dependientes de farmacias...09<br>Médico privado.....................................10<br>Otro lugar (especifique)_____________77<br>NS/NR..99<br>→	**¿Presentaba algún síntoma por el que le realizaron la prueba de detección?**<br><br>Sí.............1<br>No............2<br>→
**MUJERES CON CÓDIGO 2 EN PREGUNTA 10.B PASE A INCISO "c)"**				
a)	**prueba de Papanicolaou?**<br>*Es cuando algún médico o enfermera con la ayuda de una espátula/hisopo/cepillo extrae una muestra de células del cérvix o del cuello de su matriz y la manda al laboratorio.*	|____|____| |____|____| Meses Años<br>a1012aa a1012ab	|____|____| a1013a a1013asp	|_____| a1014a
b)	**Prueba del Papiloma Virus?**<br>*El personal de salud o usted misma introduce un cepillo pequeño para frotar el cuello de su matriz/ útero y lo coloca en un tubo con un líquido especial. Este tubo se manda a un laboratorio.*<br>MUESTRE LA TARJETA 1	|____|____| |____|____| Meses Años<br>a1012ba a1012bb	|____|____| a1013b a1013bsp	|_____| a1014b
**MUJERES CON CÓDIGO 3 EN PREGUNTA 10.B PASE A 11.01**				
c)	**Exploración clínica de los senos?**<br>*El médico o enfermera palpa los senos, para identificar cambios de coloración o de temperatura, engrosamiento de la piel o la presencia de bolitas muy duras o dolorosas.*	|____|____| |____|____| Meses Años<br>a1012ca a1012cb	|____|____| a1013c a1013csp	|_____| a1014c
d)	**Mastografía?**<br>*Se trata de una radiografía de sus senos con un aparato como el de la tarjeta que le mostraré.*<br>MUESTRE LA TARJETA 2	|____|____| |____|____| Meses Años<br>a1012da a1012db	|____|____| a1013d a1013dsp	|_____| a1014d

X. PROGRAMAS PREVENTIVOS

FILTRO: PARA MUJERES DE 20 Y MAS AÑOS

			10.16 ¿Cuál fue el resultado de su prueba?	10.17 ¿Recibió tratamiento?	10.18 ¿Cuál fue la principal causa por la que no recibío tratamiento?
			Positivo..........1<br>Negativo.........2<br>No le entregaron el resultado.........3<br>No acudió por el resultado.........4<br>NS/NR............9<br>(2, 3, 4, 9: PASE A LA SIGUIENTE PRUEBA DE DETECCIÓN)	Sí......1<br>No.....2<br>(1: PASE A LA SIGUIENTE PRUEBA DE DETECCIÓN)	No requirió tratamiento.......01<br>No hay donde atenderse.....02<br>Es caro...............................03<br>No tenía dinero...................04<br>La unidad médica le queda lejos.........................05<br>Falta de confianza.............06<br>La tratan mal…...................07<br>No tuvo tiempo…................08<br>Fue pero no lo atendieron....09<br>Por pena............................10<br>No le explicaron lo que le ban hacer……………...……..11<br>Su esposo o pareja no le permite...........................12<br>En una prueba posterior no se encontró lesión.............13<br>Otro....................................77<br>NS/NR................................99<br>PASE A SIGUIENTE PRUEBA DE DETECCIÓN, ÚLTIMA PASE A 11.01
**MUJERES CON CÓDIGO 2 EN PREGUNTA 10.B PASE A INCISO "c)"**					
a)	**prueba de Papanicolaou?**<br>*Es cuando algún médico o enfermera con la ayuda de una espátula/hisopo/cepillo extrae una muestra de células del cérvix o del cuello de su matriz y la manda al laboratorio.*	|_____|	|_____| a1016a	|_____| a1017a	|____|____| a1018a
b)	**Prueba del Papiloma Virus?**<br>*El personal de salud o usted misma introduce un cepillo pequeño para frotar el cuello de su matriz/ útero y lo coloca en un tubo con un líquido especial. Este tubo se manda a un laboratorio.*<br>MUESTRE LA TARJETA 1	|_____|	|_____| a1016b	|_____| a1017b	|____|____| a1018b
**MUJERES CON CÓDIGO 3 EN PREGUNTA 10.B PASE A 11.01**					
c)	**Exploración clínica de los senos?**<br>*El médico o enfermera palpa los senos, para identificar cambios de coloración o de temperatura, engrosamiento de la piel o la presencia de bolitas muy duras o dolorosas*	|_____|	|_____| a1016c	|_____| a1017c	|____|____| a1018c
d)	**Mastografía?**<br>*Se trata de una radiografía de sus senos con un aparato como el de la tarjeta que le mostraré.*<br>MUESTRE LA TARJETA 2	|_____|	|_____| a1016d	|_____| a1017d	|____|____| a1018d

XI. ACCIDENTES

Pregunta	Respuestas
**11.01 ¿Sufrió usted algún daño a su salud a causa de un accidente en los últimos 12 meses?** a1101	Sí......1<br>No......2 → PASE A 12.01<br>NS/NR......9 → PASE A 12.01
**11.02 ¿Cómo fue que se accidentó?** a1102<br><br>REVISE EL LISTADO DE EJEMPLOS PARA ANOTAR EL CÓDIGO	Choque entre vehículos de 4 o más ruedas......01<br>Choque en motocicleta......02 → PASE A 11.02b<br>Choque o atropellamiento como ciclista......03 → PASE A 11.02b<br>Atropellado como peatón......04 → PASE A 11.03<br>Otros accidentes de transporte: avión, tren, lancha......05 → PASE A 11.03<br>Caída a nivel de piso......06 → PASE A 11.03<br>Golpe con objeto, equipo o maquinaria......07 → PASE A 11.03<br>Caída de un nivel a otro: silla, escalera etc......08 → PASE A 11.03<br>Golpe, rasguño o mordida de persona o animal (caballo, perro etc.)......09 → PASE A 11.03<br>Exposición a corriente eléctrica, radiación......10 → PASE A 11.03<br>Exposición a humo o fuego......11 → PASE A 11.03<br>Contacto con superficies o sustancias calientes (quemaduras)......12 → PASE A 11.03<br>Exposición a fuerzas de la naturaleza: rayo, granizo......13 → PASE A 11.03<br>Picadura de animal ponzoñoso o contacto con plantas venenosas......14 → PASE A 11.03<br>Envenenamiento accidental: alimentos, medicamentos.15 → PASE A 11.03<br>Sumersión en depósitos de agua: alberca, cisterna......16 → PASE A 11.03<br>Otros accidentes que obstruyen la respiración: objeto extraño, alimento......17 → PASE A 11.03<br>Exposición accidental a otros factores......18 → PASE A 11.03<br>NS/NR......99 → PASE A 12.01
**11.02a ¿Llevaba puesto el cinturón?** a1102a	Sí......1 → PASE A 11.03<br>No......2 → PASE A 11.03
**11.02b ¿Llevaba puesto el casco?** a1102b	Sí......1<br>No......2
**11.03 ¿En qué lugar se encontraba cuando ocurrió el accidente?** a1103	Hogar......01<br>Escuela......02<br>Trabajo......03<br>Vía pública......04<br>Campo......05<br>Antro, bar......06<br>Centro recreativo o deportivo......07<br>Establecimiento comercial......08<br>Otro......77<br>NS/NR......99

XI. ACCIDENTES

11.04

¿Qué tipo de daño tuvo en ... (lea cada opción de las regiones del cuerpo)

REGIÓN DEL CUERPO

SI TUVO MÁS DE UNA LESIÓN FAVOR DE REGISTRAR LA MÁS SEVERA (POR LA QUE HAYA TENIDO QUE SER HOSPITALIZADO, INMOVILIZADO, QUE HAYA DEJADO SECUELAS O PROVOCADO DISCAPACIDAD)

Contusión.....................................01
Moretón..02
Esguince o torcedura....................03
Luxación..04
Fractura...05
Herida..06
Laceración.....................................07
Lesión de nervios..........................08
Amputación....................................09
Aplastamiento................................10
Quemadura primer grado...............11
Quemadura segundo grado..........12
Quemadura tercer grado..............13
Heridas múltiples...........................14
Daño-afectación de órganos.........15
Otras/no especificadas..................16
Ninguna lesión en esta región........17
NS/NR.........………………..............99

	REGIÓN DEL CUERPO	LESIÓN 1
a	**cara, cabeza y/o cuello?**	|____|____| a1104a
b	**tórax y/o abdomen?**	|____|____| a1104b
c	**pelvis (cadera)?**	|____|____| a1104c
d	**columna vertebral?**	|____|____| a1104d
e	**brazo y/o antebrazo?**	|____|____| a1104e
f	**mano y/o dedos de la mano?**	|____|____| a1104f
h	**muslo y/o pierna?**	|____|____| a1104h
i	**pie (tobillo) y/o dedos del pie?**	|____|____| a1104i
j	**genitales?**	|____|____| a1104j
l	**Otra (especifique)**_________________	________ a1104l a1104lsp ________

XI. ACCIDENTES

11.05 ¿Quién lo(la) atendió cuando ocurrió el accidente?

a1105

Familiar…....……......……......……......……......……......……......…......01
Amigo(a)/vecino(a)…….......……......……......……......……......…......02
Dependiente de la farmacia…….......……......……......……......…...03
Curandero......……......……......……......……......……......…...............04
Yerbero………….......……......……......……......……......…...............05
Huesero(a), sobador(a)...06
Quiropráctico...07
Homeópata.....…….......……......……......……......……......…...............08
Acupunturista...….......……......……......……......……......…...............09
Encargado de la comunidad/promotor/auxiliar de salud.10
Médico general...…......……......……......……......……......…..............11
Médico especialista...…......……......……......……......……......…....12
Dentista………….......……......……......……......……......…...............13
Enfermera...……......……......……......……......……......…..................14
Psicólogo.........……......……......……......……......……......…..............15
Otro (especifique)______ a1105esp ______77
Nadie……......……......……......……......……......……......……......20
NS/NR.............……......……......……......……......……......…...............99

11.06 ¿Cuándo sufrió el accidente estaba bajo los efectos de …

a1106

alcohol?...1
drogas?……………...2
ambos?..3
Otro (especifique) ______ a1106esp ______ 7
No estaba bajo efectos de alcohol o drogas....................8
NS/NR………………………………..…...................................9

11.07 ¿Qué problema de salud permanente le ocasionó este accidente?

a1107

Ningún problema..1
Limitación o dificultad para moverse o caminar (o lo hace con ayuda)..2
Limitación o dificultad para usar sus brazos y manos........3
Dificultad para oír...4
Dificultad para hablar..5
Dificultad para ver o ceguera..6
Otra limitación física o mental (especifique)_ a1107esp _7
NS/NR…………………………..…...................................9

XII. AGRESIÓN Y VIOLENCIA

12.01 ¿Sufrió algún daño a su salud por robo, agresión o violencia en los últimos 12 meses, incluyendo intento de suicidio? a1201

Sí	1
No	2 → PASE A 13.01
NS/NR	9 → PASE A 13.01

12.02 ¿Qué fue lo que le pasó?

REVISE EL LISTADO DE EJEMPLOS PARA ANOTAR EL CÓDIGO

PUEDE ANOTAR MÁS DE UNA OPCIÓN

Agresiones con substancias	01	a1202a
Sofocación, estrangulamiento, ahogamiento	02	a1202b
Herida por arma de fuego	03	a1202c
Herida por arma punzocortante (cuchillos, navajas, etc.)	04	a1202d
Empujón desde lugar elevado	05	a1202e
Golpes, patadas, puñetazos	06	a1202f
Agresión sexual	07	a1202g
Envenenamiento u obstrucción de las vías respiratorias por substancias u objetos calientes	08	a1202h
Agresiones verbales	09	a1202i
Otras agresiones o maltrato	76	a1202j
Otro (especifique) a1202esp	77	a1202k
NS/NR	99	a1202l

12.03 ¿Cuál fue el principal motivo? a1203

ANOTE LA OPCIÓN MÁS IMPORTANTE

Robo o asalto	01
Incidente de tránsito	02
Riña con desconocidos	03
Riña con conocidos	04
Secuestro	05
Detención	06
Violación por novio / pareja / esposo	07
Violación por algún familiar	08
Violación por desconocido	09
Violencia por algún familiar	10
Violencia por novio / pareja / esposo	11
Intento de suicidio	12
Otro (especifique) a1203esp	77
NS/NR	99

12.04 ¿En qué lugar ocurrió la agresión o violencia? a1204

Hogar	01
Escuela	02
Trabajo	03
Transporte público	04
Vía pública	05
Campo	06
Centro recreativo o deportivo	07
Antro, bar	08
Establecimiento comercial	09
Otro (especifique) a1204esp	77
NS/NR	99

XII. AGRESIÓN Y VIOLENCIA

12.06 ¿Quién lo(la) atendió cuando ocurrió la agresión?

a1206

- Familiar...01
- Amigo(a)/vecino(a)..02
- Dependiente de la farmacia..............................03
- Curandero...04
- Yerbero...05
- Huesero(a), sobador(a)...................................06
- Quiropráctico...07
- Homeópata..08
- Acupunturista...09
- Encargado de la comunidad/promotor/auxiliar de salud.10
- Médico general...11
- Médico especialista...12
- Dentista...13
- Enfermera...14
- Psicólogo..15
- Otro (especifique) a1206esp ________ 77
- Nadie...20
- NS/NR..99

12.07 Cuando sufrió la agresión o violencia ¿estaba usted bajo los efectos de …

a1207

- alcohol?...1
- drogas?..2
- ambos?...3
- Otro (especifique) a1207esp ________ 7
- No estaba bajo efectos de alcohol o drogas.......8
- NS/NR..9

12.08 ¿Qué problema de salud permanente le ocasionó la agresión o violencia?

a1208

- Ningún problema..1
- Limitación o dificultad para moverse o caminar (o lo hace con ayuda)...2
- Limitación o dificultad para usar sus brazos y manos.......3
- Dificultad para oír...4
- Dificultad para hablar.......................................5
- Dificultad para ver o ceguera..............................6
- Otra limitación física o mental (especifique) a1208esp ________ 7
- NS/NR..9

XIII. FACTORES DE RIESGO

13.01 ¿Ha fumado usted por lo menos cien cigarros (5 cajetillas) de tabaco durante toda su vida?

a1301

Sí.....1

No.....2

Nunca ha fumado.....3 → PASE A 13.10

NS/NR.....9 → PASE A 13.10

13.02 ¿Cuántos años tenía cuando fumó tabaco por primera vez, aunque fuera una sola fumada de un cigarro, de un puro o de una pipa?

a1302

SI NO SABE O SI LA RESPUESTA ES "TODA LA VIDA" O "DESDE QUE ME ACUERDO", PREGUNTE: → **¿Fue antes de los 12 años?**

SI EL(LA) ENTREVISTADO(A) CONTINÚA SIN SABER, PREGUNTE: → **¿Fue antes o después de los 20 años?**

CUANDO EL(LA) ENTREVISTADO(A) MENCIONE LA EDAD EXACTA, ANÓTELA EN LOS RECUADROS, CUANDO TENGA QUE SONDEAR ANOTE LOS CÓDIGOS CORRESPONDIENTES 112, 119 Ó 120.

Años |___|___|___|

Antes de los 12 años.....112

Antes de los 20 años.....119

Después de los 20 años.....120

NS/NR.....999

13.03 ¿Cuántos cigarros fuma actualmente?

Número de cigarros l____l____l a1303b

Frecuencia l_____l a1303a

Actualmente no fuma.....888 → PASE A 13.10

NS/NR.....999

Frecuencia:

Diario.....1

Semanal.....2

Mensual.....3

Ocasional.....4

Al menos una vez al año.....5

13.04 ¿Cuánto tiempo después de despertarse fuma su primer cigarro?

a1304

Primeros 5 minutos.....1

Entre 6 y 30 minutos.....2

Entre 31 y 60 minutos.....3

Más de 1 hora.....4

FUMADORES ACTIVOS

13.06 ¿Con qué frecuencia las advertencias en cajetillas le hacen pensar en DEJAR DE FUMAR?

a1306

Siempre.....1

Casi siempre.....2

A veces.....3

Pocas veces.....4

Nunca.....5

13.10 ¿Qué edad tenía la primera vez que tomó una bebida alcohólica en su vida?

a1310

SI NO SABE O SI LA RESPUESTA ES "TODA LA VIDA" O "DESDE QUE ME ACUERDO", PREGUNTE: → **¿Fue antes de los 12 años?**

SI EL(LA) ENTREVISTADO(A) CONTINÚA SIN SABER, PREGUNTE → **¿Fue antes o después de los 20 años?**

CUANDO EL(LA) ENTREVISTADO(A) MENCIONE LA EDAD EXACTA, ANÓTELA EN LOS RECUADROS, CUANDO TENGA QUE SONDEAR ANOTE LOS CÓDIGOS CORRESPONDIENTES 112, 119 Ó 120.

Años |___|___|___|

Antes de los 12 años.....112

Antes de los 20 años.....119

Después de los 20 años.....120

Nunca ha tomado.....000 → TERMINE ENTREVISTA

NS/NR.....999 → TERMINE ENTREVISTA

XIII. FACTORES DE RIESGO

13.11 Piense en su consumo total de alcohol. Usualmente, ¿con qué frecuencia toma usted cualquier tipo de bebida que contenga alcohol - ya sea vino, cerveza, whisky o cualquier otra bebida?

a1311

POR FAVOR NO INCLUYA
EL CONSUMO DE ALCOHOL DE PROBADITAS, SORBOS PARA COCINAR O CEREMONIAS RELIGIOSAS

Tres o más veces al día....01
Dos veces al día....02
Una vez al día....03
Casi todos los días (5-6 veces por semana)....04
Tres o cuatro veces a la semana....05
Una o dos veces a la semana....06
Dos o tres veces al mes....07
Aproximadamente una vez al mes....08
De siete a once veces al año....09
De tres a seis veces al año....10
Dos veces al año....11
Una vez al año....12
Actualmente no toma....13
NS/NR....99

13.12a Si la persona entrevistada es hombre, preguntar:

¿Con qué frecuencia toma o ha tomado 5 o más copas de cualquier bebida alcohólica en una sola ocasión?

a1312

13.12b Si la persona entrevistada es mujer, preguntar:

¿Con qué frecuencia toma o ha tomado 4 o más copas de cualquier bebida alcohólica en una sola ocasión?

UNA BEBIDA CONSISTE EN 355ml DE CERVEZA, 170ml DE VINO Ó 80ml DE RON/LICOR

A diario....01
Casi diario (5 a 6 veces por semana)....02
3 a 4 veces a la semana....03
1 a 2 veces por semana....04
2 a 3 veces al mes....05
Una vez al mes....06
7 a 11 veces al año....07
3 a 6 veces al año....08
2 veces al año....09
Una vez al año....10
Menos de una vez al año....11
Nunca....12
NS/NR....99

RESPETO A LAS PERSONAS

De acuerdo al **Artículo 13, párrafo primero del Reglamento de la Ley General de Salud en materia de Investigación para la Salud, en vigor**; "En toda investigación en la que el ser humano sea sujeto de estudio, deberá prevalecer el criterio del respeto a su dignidad y la protección a sus derechos y su bienestar."

CONFIDENCIALIDAD

Conforme a las disposiciones del **Artículo 16, del Reglamento de la Ley General de Salud en materia de Investigación para la Salud, en vigor**; "En las investigaciones en seres humanos se protegerá la privacidad del individuo sujeto de investigación, identificándolo sólo cuando los resultados lo requieran y éste lo autorice."

En referencia directa al **Artículo 38, de la Ley de Información Estadística y Geográfica en vigor,** "Los datos e informes que los particulares proporcionen para fines estadísticos o provengan de registros administrativos o civiles, serán manejados, para efectos de esta Ley, bajo la observancia de los principios de confidencialidad y reserva y no podrán comunicarse, en ningún caso, en forma nominativa o individualizada, ni harán prueba ante autoridad administrativa o fiscal, ni en juicio o fuera de él."

OBSERVACIONES

Ejemplos para las opciones de respuesta de la pregunta 11.02

V40-V79	**Choque entre vehículos de 4 o más ruedas:** incluye todos los choques, volcaduras o caídas de vehículos de cuatro o más ruedas, lesionados en la vía pública (automóvil, camioneta, minibús, autobús, transporte pesado, vehículo de rieles, vehículo sin motor y vehículo de tracción animal) 01
V20-V39	**Choque en motocicleta:** incluye a todos los pasajeros de motocicletas de dos y tres ruedas motocicleta considerar (remolque enganchado al lado de la motocicleta) que resultaron lesionados en la vía pública....02
V10-V19	**Choque o atropellamiento como ciclista:** incluye a todos los ciclistas lesionados en la vía pública (ciclista: cualquier persona que maneja un vehículo de pedal)....03
V01-V09	**Atropellado como peatón:** peatón atropellado en la vía pública por automóvil, camioneta, camión (de carga o de pasajeros), motocicleta, bicicleta, vehículo de rieles y vehículo de tracción animal (por ejemplo: carreta)....04
V80-V99	**Otros accidentes de transporte:** incluye las lesiones que ocurren a los ocupantes el medio de transporte cuando estos son acuático (por ejemplo: lancha), o aéreo (por ejemplo: avión o teleférico), o bien era terrestre pero el transporte era por tren-tranvía-metro (incluir aquí peatones atropellados por tren-tranvía-metro) ó en algún animal (por ejemplo: caballo, burro, vaca) o en vehículo traccionado-jalado por algún animal (por ejemplo: carreta). Incluir aquí peatones atropellados por tren-tranvía-metro o por algún animal o vehículo traccionado por animal. Incluya también aquí todos los casos que dude clasificar en las cuatro categorías previas....05
W00-W03 y W18	**Caída a nivel de piso:** lesiones producidas por resbalones, traspié, tropezones que suceden en el mismo nivel en el que estaba la persona antes de lesionarse. Aquí se incluyen las caídas al esquiar o al patinar sobre hielo....06
W20-W49	**Golpe con objeto, equipo o maquinaria:** golpe NO INTENCIONAL producido por un objeto lanzado o proyectado (sin intención de lastimar), o por equipo de deportes, maquinaria de agricultura, o incluso con objetos punzo-cortantes como vidrio, cuchillo, agujas u cualquier otro objeto inanimado (como maquinaria eléctrica i.e. electrodomésticos). Aquí se incluyen las lesiones producidas por balazos accidentales de pistola y/o rifle. También se incluyen las explosiones o rupturas de boiler o cilindros de gas y objetos presurizados (llantas, pipas, manguera)....07
W04-W17	**Caída de un nivel a otro:** lesiones producidas por caídas de un nivel a otro, como cuando la persona cae de un mueble (cama, mesa, sillón, silla) o de una escalera, andamio, árbol, edificio o casa habitación. Se incluye también las caídas cuando las personas lesionadas eran cargadas por otras personas....08
W50-W64	**Golpe, rasguño o mordida de persona o animal:** golpe, rasguño o mordida NO INTENCIONAL por una persona, o bien, por un animal (perro, caballo, gato, rata-ratón, animal marino, insectos, reptiles, etc.) e incluso por espinas de plantas....09
W85-W99	**Exposición a corriente eléctrica, radiación:** exposición a radiación (por ejemplo: rayos X, láser), exposición a calor o frío excesivo (por ejemplo: hielo seco), exposición a cambios en presión del aire (cambio de presión brusco en aeronaves, vista prolongada a gran altitud montañas)....10
X00-X09	**Exposición a humo o fuego:** exposición a fuego controlado y no controlado en edificio u otra construcción (edifico en llamas, chimenea, estufa, hoguera en el campo) así como exposición a material altamente inflamable (gasolina, petróleo)....11
X10-X19	**Contacto con superficies o sustancias calientes (quemaduras):** incluye las lesiones causadas por contacto con líquidos calientes (bebidas, agua, alimentos, grasas aceites) vapores y todos los objetos calientes (utensilios domésticos, máquinas, motores, herramientas etc.)....12
X30-X39	**Exposición a fuerzas de la naturaleza:** como rayo, granizo, condiciones climáticas excesivas (calor o frío), terremotos, derrumbes o avalanchas, inundaciones, tornado....13
X20-X29	**Picadura de animal ponzoñoso o contacto con plantas venenosas:** como serpientes lagartos venenosos, arañas, escorpiones, alacranes, avispas, animales y plantas marinas venenosas....14
X40-X49	**Envenenamiento accidental y exposición a sustancias nocivas:** ejemplo por alimentos, medicamentos: drogas, alcohol (etílico, butílico, etc.), exposición a gases y vapores (bióxido de azufre, oxido de nitrógeno) exposición a todos plaguicidas (insecticidas, fumigantes, fertilizante para plantas, etc.) pegamento, pinturas....15
W65-W74	**Ahogamiento y sumersión accidentales:** alberca, cisterna, bañera, aguas naturales (arroyo, lago, mar, río)....16
W75-W84	**Otros accidentes que obstruyen la respiración:** objeto extraño, alimento, sofocación accidental en cama (ropa de cama, cuerpo de la madre, etc.), confinado o atrapado en ambiente con bajo contenido de oxigeno (buce, lugares herméticos, etc.)....17
W19, X50-X57, X58-X59	**Exposición accidental a otros factores:** incluye lesiones por movimientos extraños o repetitivos, exceso de esfuerzo físico, falta de alimentación o agua (miseria, indigencia), exposición a ambientes despresurizados, y/o las lesiones que no sean especificadas claramente....18

Ejemplos para las opciones de respuesta de la pregunta 12.02

X85-X90	**Agresiones con sustancias:** como drogas, medicamentos y sustancias biológicas, agresión con sustancias corrosivas, plaguicidas, agresión con gases y vapores, agresión con sustancias productos químicos....01
X91-X92	**Agresión por sofocación:** estrangulamiento, ahogamiento y sumersión....02
X93-X95	**Arma de fuego:** agresión con disparo de arma corta, arma larga (rifle, escopeta)....03
X99	**Objetos cortantes:** puñalada (cuchillo) o cualquier otro objeto con filo: i.e. vidrio....04
Y01	**Empujón desde lugar elevado:** desde un mueble, escalera, andamio, árbol, edificio o casa habitación....05
Y04	**Golpes, patadas, puñetazos:** lucha o pelea sin armas....06
Y05	**Agresión sexual:** además de la consumación incluye el intento de violación o intento de sodomía....07
X98	**Ingestión de substancia u objetos calientes:** cualquier sustancia u objeto caliente y aquí se incluye también la ingestión de vapor....08
	**Otras agresiones o maltrato:** abuso físico, crueldad mental y tortura (Y07), negligencia, abandono (Y06). Incluir aquí también asalto con material explosivo (X96) y/o fuego, humo o flamas (X97). Incluir asalto con objetos romo o sin filo (Y00). Empujones de un solo nivel y/o empujón para que choque con objeto en movimiento (Y02). También lesiones intencionales producidas con automóvil de motor (Y03)....76
	**Otro (especifique):** cuando sea posible especificar la categoría de “otras agresiones” o maltrato” y dejar sólo la categoría de “otros” cuando no se especifique correctamente....77
