# Supplementary material for: The complex scenario of obesity, diabetes and hypertension in the area of influence of primary healthcare facilities in Mexico
Source: PLoS One. 2018 Jan 25;13(1):e0187028. doi: 10.1371/journal.pone.0187028 (PMC5784882; doi:10.1371/journal.pone.0187028)
Supplement: S4 File — (DOCX) [file pone.0187028.s004.docx]

| 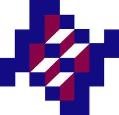Instituto Nacional de Salud Pública  Questionnaire for adults aged 18 years or more | | | | | | | | | | | | | | | | | | | |
| --- | --- | --- | --- | --- | --- | --- | --- | --- | --- | --- | --- | --- | --- | --- | --- | --- | --- | --- | --- |
| **Folio:** | | | | | | | | | | | | | | |  |  |  |  |  |
| 1. GEOGRAPHICAL IDENTIFICATION DATA | | | | | | | | | | | | | | | | | | | |
| 1.1 State or Province: 1.2 Municipality: | | | | | | | | | | | | | | | | | | | |
| 1.3 Locality: | | | | | | | | | | | | | | | | | | | |
| 2. HOUSEHOLD LOCATION DATA | | | | | | | | | | | | | | | | | | | |
| 2.1 Household address (street, avenue, drive, alley, highway, road)  2.2 No.: 2.3 Apartment/Suite No.: 2.4 Zip Code: | | | | | | | | | | | | | | | | | | | |
| 2.5 Name of the head of the family: | | | | | | | | | | | | | | | | | | | |
| 3. ADULT RESPONDENT’S DATA | | | | | | | | | | | | | | | | | | | |
| 3.1 Sex | | 3.2 Age  (years) | | | 3.3 Marital status | | | | | | 3.4 Religion | | | | | | | | |
| Male  Female | 1 |  |  |  | Single  Casado Divorced  Civil union | | | | | 1 | Catholic  Christian  Atheist  Other | | | | | | | | 1 |
|  | 2 |  |  |  |  |  |  |  |  | 2 |  |  |  |  |  |  |  |  | 2 |
| 3.Education: incomplete complete | | | | |  |  |  |  |  | 3 |  |  |  |  |  |  |  |  | 3 |
|  |  |  |  |  |  |  |  |  |  | 4 |  |  |  |  |  |  |  |  | 4 |
| Elementary school  Middle school  High school  Technical career  Bachelor’s degree  Postgraduate  No schooling | 1 | \| 2 \| \| --- \| \| 4 \| \| 6 \| \| 8 \| \| 10 \| \| 12 \| | | | 3.6 Occupation: Employee  Worker  Shopkeeper  Household worker  Unemployed  Student  Other | | | | | | | | | | | | | | 1 |
|  | 3 |  |  |  |  |  |  |  |  |  |  |  |  |  |  |  |  |  | 2 |
|  | 5 |  |  |  |  |  |  |  |  |  |  |  |  |  |  |  |  |  | 3 |
|  | 7 |  |  |  |  |  |  |  |  |  |  |  |  |  |  |  |  |  | 4 |
|  | 9 |  |  |  |  |  |  |  |  |  |  |  |  |  |  |  |  |  | 5 |
|  | 11 |  |  |  |  |  |  |  |  |  |  |  |  |  |  |  |  |  | 6 |
|  | 13 |  |  |  |  |  |  |  |  |  |  |  |  |  |  |  |  |  | 7 |
| 4. CHARACTERISTICS OF THE HOUSEHOLD MEMBERS | | | a | | b | | c* | d** | e*** | f**** | | g | h | i | | j | | k | |
|  |  |  | Age | | Sex | | Education | Occupation | Healthcare  coverage | Healthcare  facility | | Waist | Weight | Size | | Diabetes | | HBP | |
| 4.1 How many individuals aged 18 years or more live in this household? | | |  |  |  |  |  |  |  |  |  |  |  |  |  |  |  |  |  |
| Full | | | Years | | M | F |  |  |  |  |  | cm | Kg | m | | yes | no | yes | no |
| 1 | | |  |  | 1 | 2 |  |  |  |  | |  |  |  | | 1 | 2 | 1 | 2 |
| 2 | | |  |  | 1 | 2 |  |  |  |  | |  |  |  | | 1 | 2 | 1 | 2 |
| 3 | | |  |  | 1 | 2 |  |  |  |  | |  |  |  | | 1 | 2 | 1 | 2 |
| 4 | | |  |  | 1 | 2 |  |  |  |  | |  |  |  | | 1 | 2 | 1 | 2 |
| 5 | | |  |  | 1 | 2 |  |  |  |  | |  |  |  | | 1 | 2 | 1 | 2 |
| 6 | | |  |  | 1 | 2 |  |  |  |  | |  |  |  | | 1 | 2 | 1 | 2 |
| 7 | | |  |  | 1 | 2 |  |  |  |  | |  |  |  | | 1 | 2 | 1 | 2 |
| 8 | | |  |  | 1 | 2 |  |  |  |  | |  |  |  | | 1 | 2 | 1 | 2 |
| 9 | | |  |  | 1 | 2 |  |  |  |  | |  |  |  | | 1 | 2 | 1 | 2 |
| 10 | | |  |  | 1 | 2 |  |  |  |  | |  |  |  | | 1 | 2 | 1 | 2 |
| * Write the number that corresponds to the answer categories in ítem 3.5 on Education. For example: incomplete elementary school = 1; complete elementary school = 2 | | | | | | | | | | | | | | | | | | | |
| ** Employee=1; Worker=2; Shopkeeper=3; Household worker=4; Unemployed=5; Student=6; Other=7 | | | | | | | | | | | | | | | | | | | |
| *** Which social security institution provides you with coverage for health services? IMSS, ISSSTE, Seguro Popular, Pemex, Sedena, Semar, none. | | | | | | | | | | | | | | | | | | | |
| **** Health Department’s (SS) Healthcare Center=1; Health Department’s (SS) Hospital=2; IMSS=3; ISSSTE=4; PEMEX=5; Private=6; Other=7; None=8 | | | | | | | | | | | | | | | | | | | |

| 5. OVERWEIGHT AND OBESITY | | | | | | | | | | | | | | | | | |
| --- | --- | --- | --- | --- | --- | --- | --- | --- | --- | --- | --- | --- | --- | --- | --- | --- | --- |
| Please choose among the following figures:  5.1  Which of these figures is most like yours?  5.2  Which of these figures would you like to have?  5.3  Weight  kg  5.4  Size  m 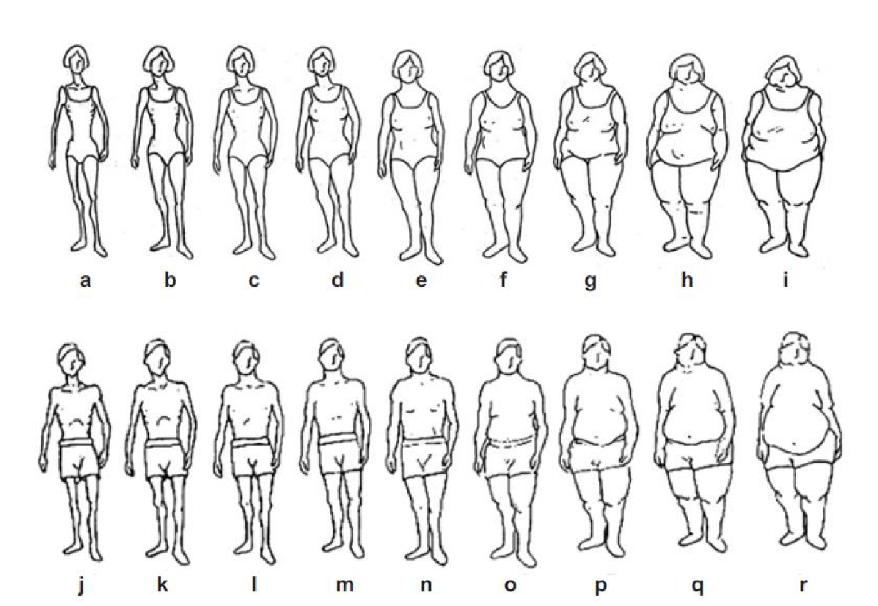 5.5 BMI | | | | | | | | | | | | | | | | | |
| **6. PREVENTIVE ASPECTS** | | | | | | | | | | | | | | | | | |
| 6.1 How often do you visit your healthcare facility? | | | | | | | | | | | 6.2 Have you received any of the following | | | | | | |
| Every week  Every two weeks Every month  Every quarter | 1 | Every six months  Every year  Not periodically  Never | | | | | | | | 5 | types of care at your Healthcare Center?  Risk-factor survey  Medical consultation for the diagnose of DM or HBP  Talks | | | | | Yes | no |
|  | 2 |  |  |  |  |  |  |  |  | 6 |  |  |  |  |  | 1 | 2 |
|  | 3 |  |  |  |  |  |  |  |  | 7 |  |  |  |  |  | 1 | 2 |
|  | 4 |  |  |  |  |  |  |  |  | 8 |  |  |  |  |  | 1 | 2 |
| 6.3 Have they spoken to you about diabetes? | | 6.4 Have they told you why this disease occurs? | | 6.5 Have they explained to you how to prevent it? | | | | | | | 6.6 Have you had screening tests for it? | | **If your last answer was YES** | | | | |
|  |  |  |  |  |  |  |  |  |  |  |  |  | 6.7 Can you tell us which? | | | | |
| Yes  No  I don’t remember | 1 | Yes  No  I don’t remember | 1 | Yes  No  I don’t remember | | | | | | 1 | Yes  No  I don’t remember | 1 |  |  |  |  |  |
|  | 2 |  | 2 |  |  |  |  |  |  | 2 |  | 2 |  | | | | |
|  | 3 |  | 3 |  |  |  |  |  |  | 3 |  | 3 |  | | | | |
| 6.8 Have they spoken to you about high blood pressure? | | 6.9 4 Have they told you why this disease occurs? | | 6.10 Have they explained to you how to prevent it? | | | | | | | 6.11 Have you had screening tests for it? | | **If your last answer was YES** | | | | |
|  |  |  |  |  |  |  |  |  |  |  |  |  | 6.12 Can you tell us which? | | | | |
| Yes  No  I don’t remember | 1 | Yes  No  I don’t remember | 1 | Yes  No  I don’t remember | | | | | | 1 | Yes  No  I don’t remember | 1 |  |  |  |  |  |
|  | 2 |  | 2 |  |  |  |  |  |  | 2 |  | 2 |  | | | | |
|  | 3 |  | 3 |  |  |  |  |  |  | 3 |  | 3 |  | | | | |
| **7. PHYSICAL ACTIVITY** | | | | | | | | | | | | | | | | | |
| Think of all the **vigorous** activities that you carried out in the **last 7 days** (Vigorous activities are those that require a **strong physical effort** and make you breathe **much more loudly** than normal). Think only of those activities that you carried out uninterruptedly for at least 10 minutes. | | | | | | | | | | | | | | | | | |
| 7.1 During the **last 7 days**, how many days did you carry out **vigorous** physical activities such as lifting heavy objects, shoveling, doing aerobics or pedal quickly on a bicycle? | | | | | | | | | | | 7.2 For how long, total, did you usually carry out **vigorous** physical activities on one of the days when you carried them out? | | | | | | |
| Number of days per week | | | | 1 | 2 | 3 | 4 | 5 | 6 | 7 | Hours per day  Minutes per day  I don’t know/I’m not sure | | |  |  |  |  |
| If you did **NOT** carry out any vigorous physical activity, move on to question 7.3 | | | | | | | | | | |  |  |  |  |  |  |  |
|  |  |  |  |  |  |  |  |  |  |  |  |  |  | 9 | 9 | 9 | 9 |

Think of all the moderate activities that you carried out in the **last 7 days** (Moderate activities are those that require a **moderate physical effort** and make you breath **slightly deeper** than normal). Consider *only* those activities that you carried out continually for at least 10 minutes.

7.3 During the **last 7 days**, for how many days did you carry out **moderate** 7.4 Usually, how much time do you devote on any of those days to physical activities, such as carrying light objects, pedaling on a bicycle at a carrying out **moderate** physical activities?

regular pace, playing soccer or basketball? Do not include walks

Hours per day

Number of days per week 1 2 3 4 5 6 7 Minutes per day

If you did **NOT** carry out any moderate physical activity, move on I don’t know/I’m not sure

to question 7.5

Think of the time that you devoted to walking during the **last 7 days**. This includes **housework**, **walks** from one place to another, or **any other walk** that you may have carried out for recreational purposes, as a sport or exercise or for pleasure.

7.5 During the **last 7 days**, how many days did you walk for at least 10 Hours per day

minutes? Minutes per day

Number of days per week 1 2 3 4 5 6 7 I don’t know/I’m not sure

If you do **NOT** walk, move on to question 7.7 9 9 9 9

The last question refers to the **time that you remained sitting** during the week during the **last 7 days**. Include sitting time at work, home, studying and in your spare time. This may include time sitting at a desk, visiting friends, reading or sitting or **lying down** while watching television.

7.7 During the **last 7 days**, how long did you remain **sitting** in one day Hours per day

of the week? Minutes per day

I don’t know/I’m not sure 9 9 9 9

1. SMOKING
   1. Do you smoke cigarettes, pipe, cigars or any other tobacco product? 8.3 How many cigarettes do you smoke?

Yes 1 One 1

No **(move on to question 9.1)** 2 Two 2

No answer **(move on to question 9.1)** 3 Three 3

- 1. How often do you smoke? From 4 to 6 4

7 to 10 5 per day 1 A pack 6

Every other day 2 Two packs 7

Twice a week 3 More than two packs 8

Once a week 4 8.4 Have you sought help to quit smoking?

Once a month 5 Yes 1

Occasionally 6 No 2

1. MEALS
   1. Who prepares the meals at your home? 9.2 Where do you eat breakfast? 9.3 Where do you eat lunch?

At home 1 At home 1

Mother 1 Outdoors 2 Outdoors 2

Father 2 I do not eat breakfast 3 I do not eat lunch 3

Grandparent 3 9.4 Where do you eat dinner? 9.5 Where do you eat supper?

Son or daughter 4 At home 1 At home 1

Some other family member 5 Outdoors 2 Outdoors 2

Other 6 I don’t eat dinner 3 I don’t eat supper 3

<INSERTAR CONTENIDO DEL ARCHIVO “Cuestionario Demanda – Tabla de alimentos a Pregunta 10.4”>

| 10.5 During the last 12 months, what tests did your physician request to monitor your sugar levels? (you may mark several) | Glycosylated hemoglobin |  | |  | | |  | | | |  | | |  | | |
| --- | --- | --- | --- | --- | --- | --- | --- | --- | --- | --- | --- | --- | --- | --- | --- | --- |
|  | Blood glucose |  | |  | | |  | | | |  | | |  | | |
|  | Blood glucose test strip |  | |  | | |  | | | |  | | |  | | |
|  | General urine test |  | |  | | |  | | | |  | | |  | | |
|  | Urine proteins |  | |  | | |  | | | |  | | |  | | |
|  | Urine strip |  | |  | | |  | | | |  | | |  | | |
|  | Self-monitoring |  | |  | | |  | | | |  | | |  | | |
|  | None |  | |  | | |  | | | |  | | |  | | |
| 10.6 During the last 6 months, what preventive measures have you taken? (you may mark several) | Retinal examination |  | |  | | |  | | | |  | | |  | | |
|  | Visual acuity test |  | |  | | |  | | | |  | | |  | | |
|  | Foot examination test |  | |  | | |  | | | |  | | |  | | |
|  | Overall urine test |  | |  | | |  | | | |  | | |  | | |
|  | Daily Aspirin |  | |  | | |  | | | |  | | |  | | |
|  | Other |  | |  | | |  | | | |  | | |  | | |
|  | None |  | |  | | |  | | | |  | | |  | | |
| 10.7 Have you had any (or more than one) of the following issues due to diabetes? | Ulcers in legs/feet |  | |  | | |  | | | |  | | |  | | |
|  | Insensitive feet |  | |  | | |  | | | |  | | |  | | |
|  | Amputation |  | |  | | |  | | | |  | | |  | | |
|  | Impaired vision |  | |  | | |  | | | |  | | |  | | |
|  | Damaged retina |  | |  | | |  | | | |  | | |  | | |
|  | Vision loss |  | |  | | |  | | | |  | | |  | | |
|  | Dialysis |  | |  | | |  | | | |  | | |  | | |
|  | Heart attack |  | |  | | |  | | | |  | | |  | | |
|  | Diabetic coma |  | |  | | |  | | | |  | | |  | | |
| **11. QUESTIONS ABOUT HIGH BLOOD PRESSURE** | | | | | | | | | | | | | | | | |
| 11.1 Does anyone in your family suffer from hypertension?  Yes No | | | | | 1 | If your answer is **NO**, move on to question 11.4; if your answer | | | | | | | | | | |
|  |  |  |  |  | 2 | is **YES**, move on to question 11.2 | | | | | | | | | | |
| 11.Write the names of those family members who live with hypertension | | | | | | | | | | | | | 11.3 Since when? | | | |
| 1 | | | | | | | | | | | | |  | | | |
| 2 | | | | | | | | | | | | |  | | | |
| 3 | | | | | | | | | | | | |  | | | |
| 4 | | | | | | | | | | | | |  | | | |
| 5 | | | | | | | | | | | | |  | | | |
| **If you are a female, move on to question 11.4; if you are a male, move on to question 11.6** | | | | | | | | | | | | | | | | |
| 11.4 Did a physician diagnose high blood pressure during your pregnancy? | | | 11.5 Has a physician or a healthcare staff member diagnosed you with preeclampsia? | | | | | | | 11.6 Do you currently take medication to control your blood pressure? | | | | | | |
| Yes  No  I have never been pregnant | | 1 |  |  |  |  |  |  |  |  |  |  |  |  |  |  |
|  |  | 2 | Yes  No | | | | | | 1 | Yes  No | | | | | | 1 |
|  |  | 3 |  |  |  |  |  |  | 2 |  |  |  |  |  |  | 2 |
| 11. Write the names of those members of your family who suffer from high blood pressure and the facility to which they go for health care (you may mark several options) | | | | | | | | SS  HCC | | SS Hosp | IMSS | ISSSTE | Pemex | Private | None | |
| 1 | | | | | | | |  | |  |  |  |  |  |  | |
| 2 | | | | | | | |  | |  |  |  |  |  |  | |
| 3 | | | | | | | |  | |  |  |  |  |  |  | |
| 4 | | | | | | | |  | |  |  |  |  |  |  | |
| 5 | | | | | | | |  | |  |  |  |  |  |  | |

| 11.8 What kind of treatment do they receive? (you may mark more than one option) | | | | | | | | | | | | |
| --- | --- | --- | --- | --- | --- | --- | --- | --- | --- | --- | --- | --- |
| Names of relatives with high blood pressure under treatment | | | Pills | | Homeo_ pathy | | Herbal medicine | Diet (low-salt) | Traditional medicine | Exercise | None | |
| 1 | | |  | |  | |  |  |  |  |  | |
| 2 | | |  | |  | |  |  |  |  |  | |
| 3 | | |  | |  | |  |  |  |  |  | |
| 4 | | |  | |  | |  |  |  |  |  | |
| 5 | | |  | |  | |  |  |  |  |  | |
| 11.9 In the last year, how often did you take your blood pressure? | | | | | | | | | | | | |
| Daily Weekly Monthly Quarterly Twice a year Occasionally Never 7  1  2  3  4  5  6 | | | | | | | | | | | | |
| 12. CARDIOVASCULAR DISEASE | | | | | | | | | | | | |
| 12.1 Have you ever experienced strong pain in your chest, difficulty to breathe, palpitations or a great discomfort for half an hour or longer? | | 12.2 Has your physician told you that you had … | | | | | | | | | | |
|  |  | A heart attack?  Angina pectoris?  Heart failure?  Any other heart disease? | | | | | | | | | | 1 |
|  |  |  |  |  |  |  |  |  |  |  |  | 2 |
| Yes  No | 1 |  |  |  |  |  |  |  |  |  |  | 3 |
|  | 2 |  |  |  |  |  |  |  |  |  |  | 4 |
| 13. HYPERLIPIDEMIA AND NEUROVASCULAR DISEASE | | | | | | | | | | | | |
| 13.1 Have you been tested for blood cholesterol? | | | | 13.2 Have you evere been tested for blood triglycerides? | | | | | | | | |
| Yes, it was found to be normal  Yes, it was found to be high  No | | | 1 | Yes, they were found to be normal  Yesthey were found to be high  No | | | | | | | | 1 |
|  |  |  | 2 |  |  |  |  |  |  |  |  | 2 |
|  |  |  | 3 |  |  |  |  |  |  |  |  | 3 |
| 13.3 In the last 12 months, did you receive any treatment for high cholesterol?  (you may mark more than one option) | | | | | | | 13.4 Have you ever had a vascular cerebral event? (cerebral infarction, cerebral embolism, stroke) | | | | | |
| Pravastatin, simvastatin, atorvastatin, rosuvastatin or fluvastatin  Reducing the intake of fats and cholesterol in foods  Increasing physical activity  None | | | | | | 1 |  |  |  |  |  |  |
|  |  |  |  |  |  | 2 | Yes  No  I don’t know/remember | | | | | 1 |
|  |  |  |  |  |  | 3 |  |  |  |  |  | 2 |
|  |  |  |  |  |  | 4 |  |  |  |  |  | 99 |
| **14. FAMILY HISTORY** | | | | | | | | | | | | |
| 14.1 Does your mother have diabetes or high blood sugar levels? | | | 14.7 1 Does your father have diabetes or high blood sugar levels? | | | | | | | | | |
| Yes  No **(move on to question 14.3)**  I don’t know/remember | | 1 | Yes  No **(move on to question 13.9)**  I don’t know/remember | | | | | | | | | 1 |
|  |  | 2 |  |  |  |  |  |  |  |  |  | 2 |
|  |  | 99 |  |  |  |  |  |  |  |  |  | 99 |
| 14.2 At what age was your mother diagnosed with diabetes? years | | | 14.8 At what age was your father diagnosed with diabetes? years | | | | | | | | | |
| 14.3 Did your mother suffer from hypertension or high blood pressure? | | | 14.9 Did your father suffer from hypertension or high blood pressure? | | | | | | | | | |
| Yes  No **(move on to question 14.5)**  I don’t know/remember | | 1 | Yes  No **(move on to question 14.11)**  I don’t know/remember | | | | | | | | | 1 |
|  |  | 2 |  |  |  |  |  |  |  |  |  | 2 |
|  |  | 99 |  |  |  |  |  |  |  |  |  | 99 |
| 14.4 At what age was your mother diagnosed with hypertension?  years | | | 14.10 At what age was your father diagnosed with hypertension?  years | | | | | | | | | |
| 14.5 Did your mother have a heart attack ? | | | 14.11 Did your father have a heart attack ? | | | | | | | | | |
| Yes  No **(move on to question 14.7)**  I don’t know/remember | | 1 | Yes  No **(move on to section 15)**  I don’t know/remember | | | | | | | | | 1 |
|  |  | 2 |  |  |  |  |  |  |  |  |  | 2 |
|  |  | 99 |  |  |  |  |  |  |  |  |  | 99 |
| 14.6 At what age did your mother have her first heart attack? years | | | 14.12 At what age did your father have his first heart attack? years | | | | | | | | | |

| **15. HEALTHCARE QUALITY** | | | | | | |
| --- | --- | --- | --- | --- | --- | --- |
| 15.1 Do you go regularly to the healthcare center to receive care? | | 15.2 At what time do you leave your home to go to the healthcare center for a consultation?  __________________________ | | 15.3 How long does it take you to get to the healthcare center? | | |
|  |  |  |  | Less than 15 minutes  Between 16 and 30 minutes  Between 30 minutes and 1 hour  More than 1 hour | | 1 |
| Yes  No  I don’t remember | 1 |  |  |  |  | 2 |
|  | 2 |  |  |  |  | 3 |
|  | 99 |  |  |  |  | 4 |
| 15.4 How long do you have to wait once you arrive at the healthcare center?  Less than 10 minutes  Less than 30 minutes  Less than 1 hour  Between one and two hours  More than two hours | | 1 | 15.5 Do you schedule an appointment Yes  before you go to the healthcare center? No | | | 1 |
|  |  |  |  |  |  | 2 |
|  |  | 2 | 15.6 How much time do you spend in the medical consultation? __________ min | | | |
|  |  | 3 |  |  |  |  |
|  |  | 4 | 15.7 Does the doctor examine you? Yes  No | | | 1 |
|  |  | 5 |  |  |  | 2 |
| 15.8 Which of these actions are performed  on you at the healthcare center? (you may mark several) | | | 15.9 Are you provided with all your medications at the healthcare center? | | 15.10 What do you do when not all your medications are provided to you? | |
|  |  |  |  |  | You buy them  You wait until they are available  You get them through friends  None | 1 |
|  |  |  | Yes  No  I don’t know/remember | 1 |  | 2 |
| You are measured  You are weighted  Your blood pressure is taken  You are prescribed medication  Laboratory tests are performed on you  You are explained about your treatment  Your health issue is discussed with you  None | | 1 |  | 2 |  | 3 |
|  |  | 2 |  | 3 |  | 4 |
|  |  | 3 | 15.11 How does the nurse treat you? | | 15.12 How does the physician treat you? | |
|  |  | 4 | Lovingly  With indifference  With respect  With patience  Disrespectfully | 1 | Lovingly  With indifference  With respect  With patience  Disrespectfully | 1 |
|  |  | 5 |  | 2 |  | 2 |
|  |  | 6 |  | 3 |  | 3 |
|  |  | 7 |  | 4 |  | 4 |
|  |  | 8 |  | 5 |  | 5 |
| **THANK YOU, WE GREATLY APPRECIATE YOUR PARTICIPATION** | | | | | | |
